# The WRKY transcription factor superfamily: its origin in eukaryotes and expansion in plants

**DOI:** 10.1186/1471-2148-5-1

**Published:** 2005-01-03

**Authors:** Yuanji Zhang, Liangjiang Wang

**Affiliations:** 1 Plant Biology Division, The Samuel Roberts Noble Foundation, Ardmore, OK 73402, USA

## Abstract

**Background:**

WRKY proteins are newly identified transcription factors involved in many plant processes including plant responses to biotic and abiotic stresses. To date, genes encoding WRKY proteins have been identified only from plants. Comprehensive search for WRKY genes in non-plant organisms and phylogenetic analysis would provide invaluable information about the origin and expansion of the WRKY family.

**Results:**

We searched all publicly available sequence data for WRKY genes. A single copy of the WRKY gene encoding two WRKY domains was identified from *Giardia lamblia*, a primitive eukaryote, *Dictyostelium discoideum*, a slime mold closely related to the lineage of animals and fungi, and the green alga *Chlamydomonas reinhardtii*, an early branching of plants. This ancestral WRKY gene seems to have duplicated many times during the evolution of plants, resulting in a large family in evolutionarily advanced flowering plants. In rice, the WRKY gene family consists of over 100 members. Analyses suggest that the C-terminal domain of the two-WRKY-domain encoding gene appears to be the ancestor of the single-WRKY-domain encoding genes, and that the WRKY domains may be phylogenetically classified into five groups. We propose a model to explain the WRKY family's origin in eukaryotes and expansion in plants.

**Conclusions:**

WRKY genes seem to have originated in early eukaryotes and greatly expanded in plants. The elucidation of the evolution and duplicative expansion of the WRKY genes should provide valuable information on their functions.

## Background

Transcriptional control is a major mechanism whereby a cell or organism regulates its gene expression. Sequence-specific DNA-binding transcription regulators, one class of transcription factors [1], play an essential role in modulating the rate of transcription of specific target genes. In this way, they direct the temporal and spatial expressions necessary for normal development and proper response to physiological or environmental stimuli. Comparative genome analysis reveals that genes for transcription regulators are abundantly present in plant and animal genomes, and the evolution and diversity of eukaryotes seem to be related to the expansion of lineage-specific transcription regulator families [2].

WRKY proteins are recently identified transcriptional regulators comprising a large gene family [3]. The first cDNA encoding a WRKY protein, SPF1, was cloned from sweet potato (*Ipomoea batatas*) [4]. Numerous genes for WRKY proteins have since been experimentally identified from more than 10 other plant species, including *Arabidopsis thaliana *[5,6], wild oats (*Avena fatua*) [7], orchardgrass (*Dactylis glomerata*) [8], barley (*Hordeum vulgare*) [9], tobacco (*Nicotiana tabacum*) [10-13], chamomile(*Matricaria chamomilla*) [14], rice (*Oryza sativa*) [9,15], parsley (*Petroselinum crispum*) [16,17], a desert legume (*Retama raetam*) [18], sugarcane (*Saccharum *hybrid cultivar) [19], bittersweet nightshade (*Solanum dulcamara*) [20], potato (*Solanum tuberosum*) [21,22], and wheat (*Triticum aestivum*) [9]. In addition, over 70 WRKY genes were identified in the Arabidopsis genome by sequence similarity comparisons [2,23]. To date, WRKY genes have not been cloned from species other than plants. The absence of WRKY homologues in the genomes of animals (*Caenorhabditis elegans *and *Drosophila melanogaster*) and yeast (*Saccharomyces cerevisiae*) [2] leads to the suggestion that WRKY transcription regulators are restricted to the plant kingdom [2,3]. As genome sequence data for species representing several major eukaryotic lineages are now available, we can re-examine whether WRKY genes are plant-specific or have ancestors predating the appearance of plants.

The WRKY family proteins contain one or two highly conserved WRKY domains characterized by the hallmark heptapeptide WRKYGQK and a zinc-finger structure distinct from other known zinc-finger motifs [3]. To regulate gene expression, the WRKY domain binds to the W box in the promoter of the target gene to modulate transcription [5,7,16,24]. In addition to the W box, a recent study indicates that the WRKY domain can also bind to SURE, a sugar responsive *cis *element, as a transcription activator [9].

In plants, many WRKY proteins are involved in the defense against attack from pathogenic bacteria [6,22,23,25-27], fungi [26], viruses [12,26,28], and oomycetes [21,26,29]. Further, WRKY genes are implicated in responses to the abiotic stresses of wounding [11,30], the combination of drought and heat [31], and cold [18,20]. It is also evident that some members of the family may play important regulatory roles in morphogenesis of trichomes [32] and embryos [8], senescence [26,33-35], dormancy [18], plant growth [27], and metabolic pathways [7,9,32,36].

Based on the number of WRKY domains and the pattern of the zinc-finger motif, Eulgem et al. [3] classified members of the WRKY superfamily from the Arabidopsis genome into three groups. Members of Group 1 typically contain two WRKY domains, while most proteins with one WRKY domain belong to Group 2. Group 3 proteins also have a single WRKY domain, but the pattern of the zinc-finger motif is unique. Eulgem et al. [3] further divided Group 2 into five subgroups, according to the phylogenetic analysis of the WRKY domains.

Given the large family of WRKY genes with divergent regulatory functions in important plant processes, it would be desirable to understand the evolutionary origin and gene duplications leading to the multi-member WRKY family. The clarification of the phylogenetic relationships among WRKY genes in model plants will also assist understanding of the functions of these genes in important crops. We have comprehensively searched all currently available sequence data for the existence of WRKY genes outside the plant kingdom. Homologues of WRKY genes are found from two eukaryotic species: *Giardia lamblia*, a primitive protozoan, and *Dictyostelium discoideum*, a slime mold. The data indicate an early origin of WRKY genes in eukaryota and tremendous gene amplifications in the plant lineage. We then cataloged the WRKY genes from the rice genome and compared them with Arabidopsis WRKY genes. We also identified WRKY genes from expressed sequence tags (ESTs) and EST-assembled sequence contigs from nineteen plant species. The result suggests that WRKY gene duplication events correlate with the increasing structural and functional complexities in land plants. We propose a model for the evolution of WRKY genes.

## Results

### WRKY genes in non-plant eukaryotes

We searched for WRKY genes in two comprehensive datasets, GenBank's non-redundant (nr) and dbEST of all species. Together these datasets contain over 13 million sequence records from more than 110,000 organisms [37]. Homologues of WRKY proteins are not found in the superkingdoms of archaea and eubacteria. In eukaryotes, no WRKY genes are identified from the lineages of fungi and animals.

Interestingly, two WRKY homologues were identified from non-plant eukaryotic species, and both have two WRKY domains [see Additional files 1 and 2]. The first protein (GenBank accession: EAA40901) is encoded by an intronless gene in the draft genome sequence of *Giardia lamblia *[38]. The unicellular protist *Giardia *is one of the most primitive organisms that represent the earliest branching among extant eukaryotes [39,40]. The second (accession AAO52331) is encoded by the genomic sequence of chromosome 2 of the slime mold *Dictyostelium discoideum *[41]. The genomic sequence for the WRKY domains were assembled from sequences generated from three libraries prepared by two groups [42], indicating that it is not from sequence contamination. The gene contains an intron, which interrupts the coding region between the two WRKY domains. For this species, about 150,000 EST sequences are currently available in GenBank. One EST (accession AU033476) aligns to the WRKY gene, indicating that the gene is expressed. *D. discoideum *belongs to the Mycetozoa, a lineage more closely related to animals and fungi than to green plants [41,43].

### A WRKY gene in a green alga

*Chlamydomonas reinhardtii *is a unicellular green alga with a cell wall. It also has chloroplasts for photosynthesis. The evolutionary position of the species is located before the divergence of land plants [44,45]. The release 1.0 of its genome sequence has approximately 9 × whole genome shotgun coverage [46]. Since the gene annotation for the release is still at a preliminary stage, we predicted WRKY genes from the genome sequence (see Methods). The sequence similarity search between the genome sequence and Pfam's WRKY domain sequences indicated that the sequence 'Scaffold_1387' may encode WRKY domains. This sequence was then used for further WRKY domain and gene predictions. Despite minor differences in the gene structure prediction, both gene prediction programs FGENESH and GENSCAN agree on the major features of the protein, including the presence of two WRKY domains [see Additional files 1 and 2]. Moreover, the predicted peptide sequence of the WRKY domains is identical among all the gene and domain predictions. Sequence alignment by blastn indicates that six ESTs are from the predicted coding regions of the gene; the GenBank accessions for these ESTs are BI727288, AW772895, BM000804, BG846749, BE121978 and BQ821537.

### A catalog of WRKY genes in rice

Rice, one of the most important crops for world agriculture, is recognized as a model monocot for the study of cereal crop genomes. A comprehensive catalog of rice WRKY genes would provide a basis for investigating the evolutionary patterns of the gene family and for transferring knowledge of the functions of these transcription factors from Arabidopsis to rice and from rice to other cereal crops.

We identified the members of the WRKY family in rice (Japonica variety) from its published genome sequence [47]. The WRKY gene identification procedure employed in this study (see Methods) was first tested with the Arabidopsis genome sequence. The procedure successfully identified all reported Arabidopsis WRKY genes [3,23]. The rice genome seems to encode 109 WRKY proteins, four of which have incomplete WRKY domains. The remaining 105 proteins with complete WRKY domains, listed in Additional file 3, were used for further analysis. The multiple sequence alignment of WRKY domains from rice, Arabidopsis, the green alga, the slime mold and *Giardia lamblia*, and the conserved WRKY domain patterns can be found in Additional file 2. Some rice genes encode identical WRKY domains. For example, OsWRKY34 and OsWRKY57 share identical amino acid sequences in the WRKY domains, but the nucleotide sequences for the domains are not identical and they are located in different chromosomes (1 and 4, respectively), indicating that they are distinct genes. Similarly, OsWRKY8 located in Chromosome 6 and OsWRKY76 located in Chromosome 2 also represent two genes. The following genes in parenthesis share the identical WRKY domains and have a high identity of the corresponding coding nucleotide sequences: (OsWRKY9, 101), (12, 98 and 99), (21, 97), (29, 96), (39, 105), (51, 103), (73, 104), (80, 102), and (82, 100). These highly similar genes may represent newly duplicated paralogues. The 105 genes are unevenly distributed in the 12 chromosomes, ranging from 25 genes (the highest number) in Chromosome 1 to two genes (the lowest) in Chromosome 10. Sequence alignment indicates that 60 WRKY genes have one or more matched rice ESTs from the dbEST database (data not shown). Out of the 105 proteins, 13 have two WRKY domains. We assigned the WRKY domains into subfamilies using phylogenetic analysis with already classified AtWRKY genes from *A. thaliana *[3] as the reference. Eleven proteins with two WRKY domains are assigned to Group 1 because their C-terminal domains belong to this group. Since the N- and C-terminal domains form distinct clusters, we designated the two domains as 1N and 1C, respectively. Six proteins with a single domain also belonged to Group 1. While OsWRKY15, 16, 73 and 104 have a single domain homologous to Group 1N, OsWRKY13 and 91 contain a single Group 1C domain. Interestingly, both N- and C-domains of the other two double-domain-containing proteins (OsWRKY66 and 67) are always clustered with Group 3 domains. Thirty-five single WRKY domain proteins are also assigned to this group. All together, there are 39 domains or 37 proteins in Group 3. We assigned 49 proteins to three new groups, Group 2_a + 2_b (13), Group 2_c (18), and Group 2_d + 2_e (18). These new groups are reorganized from the five subgroups IIa through IIe in Eulgem et al. [3] (see details of the classification in Discussion). Domains of OsWRKY 25 and 95 cannot be consistently classified and therefore remain unassigned [see Additional file 3].

Interestingly, several variant patterns of the WRKY domains exist in the rice WRKY proteins. Although the WRKYGQK peptide is highly conserved, nine variants with one or two amino acids substituted are observed in 19 domains, most of which belong to Groups 3 and 2_c (Table 1). While WRKYGEK and WRKYGKK are two common variants shared by seven (all in Group 3) and five (all in Group 2_c) domains, respectively, each of the other seven different heptapeptides occurs in only one protein. The WRKY domains also contain patterns of zinc-finger motifs that have not been reported in the literature (Table 1). No variants are found in domains of Groups 1C and 2_a + 2_b. The WRKY genes encoding the variant domain patterns might be functional, because 10 genes with a total of seven heptapeptide variants and two zinc-finger motif variants have sequenced ESTs, although the DNA binding capacity may be reduced [48]. Furthermore, ESTs have been sequenced from the gene regions for the variants of WRKYGEK, WRKYGKK, WKKYGQK and C_X6_C_X28_H_X1_C, indicating that these patterns are not artifacts of the gene prediction (Table 1).

**Table 1:** Variants of the conserved WRKYGQK peptide and zinc-finger motifs in rice WRKY domains

Pattern	Domain	Group	Available ESTs	
			
			ID^a^	Encoding the domain
*Variants of WRKYGQK*				
WRKYG**E**K	OsWRKY7	3		
	OsWRKY8	3	CA755335	Yes
	OsWRKY65	3		
	OsWRKY72	3	CF282152, CF330819, CF303772, CF282153, CF330818, CF305084, CF328161	Yes
	OsWRKY76	3	CA755335	
	OsWRKY77	3		Yes
	OsWRKY94	3		
WRKYG**K**K	OsWRKY20	2_c		
	OsWRKY27	2_c		
	OsWRKY36	2_c	D43156	No
	OsWRKY46	2_c	TC154521, AU093050	No
	OsWRKY63	2_c	TC143003, BE230596, BM419201	Yes
WR**IC**GQK	OsWRKY15	1N		
WR**MC**GQK	OsWRKY16	1N		
W**K**KYGQK	OsWRKY25	unassigned	AU162739	Yes
W**I**KYGQK	OsWRKY55	3		
W**KR**YGQK	OsWRKY66C	3	AW155482	No
W**S**KY**E**QK	OsWRKY67N	3	CA760141	No
WRKY**SE**K	OsWRKY92	3		
*Variants of zinc-finger motifs*				
C_X5_C_X25_H_**X2**_C	OsWRKY6	2_d + 2_e		
C_**X8**_C_X25_H_X1_C	OsWRKY67N	3	CA760141	No
C_**X6**_C_X28_H_X1_C	OsWRKY68	3	TC103502	Yes

^a^TIGR's TCs or GenBank's accessions.

### Survey of WRKY genes in land plants

Since the genomes of rice and Arabidopsis have numerous WRKY genes whereas the green alga may have only a single copy, it would be interesting to investigate the gene duplication events of WRKY family during the course of evolution from unicellular plant organisms to flowering plants and the relationship between expansion of the WRKY family and the increased structural and functional complexities of the higher plants. Ideally, the complete set of WRKY genes should be identified from species representing different branches on the evolutionary tree of plants for further analysis. Unfortunately, genome sequence is currently not available for most plant species. However, a large number of EST sequences for many plants are publicly available and can be used to roughly estimate the minimum number of WRKY genes in these species.

We first surveyed GenBank's dbEST set and found that WRKY genes are widespread in land plants, as over 40 species have expressed WRKY genes (data not shown). We then estimated the number of unique WRKY genes for 17 species using their Gene Indices, which are assembled EST sequence contigs with the minimal redundancy, provided by The Institute for Genomic Research (TIGR) [49]. The analysis also included ESTs for the moss *Physcomitrella patens *and the fern *Ceratopteris richardii *whose Gene Indices are not available [see Additional file 4]. For the EST set, redundant ESTs for WRKY proteins were manually removed. Together these 19 species represent different branches on the evolutionary tree of the land plants. While the moss Physcomitrella is an early diverged land plant, the fern is an ancient vascular plant. The conifer *Pinus *represents the gymnosperm lineage, and the remaining are the evolutionarily more advanced flowering plants [50].

ESTs encoding WRKY proteins were identified in all the 19 species. Moreover, multiple WRKY genes are represented in the EST or contig sets for most plants including the moss and pine, with the most WRKY genes (109) from soybean [see Additional file 4]. Although the actual number of WRKY genes encoded in a plant genome can only be known using the genome sequence, EST datasets are useful to estimate the relative size of WRKY family in plant species whose genome sequences are not available, given sufficient large EST sets sampled from the genomes. If a set of ≥ 50,000 ESTs is considered a large sample, then pine, moss and 12 flowering plants listed in Additional file 4 have enough ESTs for the estimation. The comparison of the number of WRKY genes identified from EST sets with comparable size suggests that the genomes of moss and pine seem to encode much fewer WRKY genes than evolutionarily advanced flower plants. We also compared pine with Arabidopsis in another analysis using ESTs from GenBank's dbEST database (as of 10/28/2002). We identified ESTs for 46 Arabidopsis WRKY genes but only two pine WRKY genes, although Arabidopsis' EST set (176,915) is less than three times bigger than pine's (60,226).

The abundance of WRKY ESTs in the total EST set is lower for pine, fern and moss than for flowering plants, as the percentage of WRKY ESTs in the total EST set for the three non-flowering plants is among the lowest [see Additional file 4]. The WRKY EST abundance in an EST dataset may be affected by the number of WRKY genes in the species and by the expression levels of WRKY genes in the cells from which ESTs were obtained. For example, WRKY EST abundance for pine is much lower than that for tomato (0.0086% : 0.3546%, or ~ 1 : 40). The low WRKY EST abundance of pine may be partly due to fewer WRKY genes from pine than from tomato (4 : 51, or ~ 1 : 13) [see Additional file 4]. It is also possible that pine WRKY genes are lowly expressed. For example, for a tomato WRKY gene the average EST count is > 10, but for pine it is < 2.

The identified WRKY genes were phylogenetically classified into five groups [see Additional file 4]. In six WRKY genes identified from the moss ESTs, two are homologous to Group 2_c and three belong to Group 2_d + 2_e, indicating an early origin of these groups in land plants. In comparison, genes in Group 3 are only identified in the EST sets of flowering plants but not from EST data of more ancient plants, i.e., moss, fern and pine [see Additional file 4].

### Phylogeny of the WRKY domains

**Figure 1:**
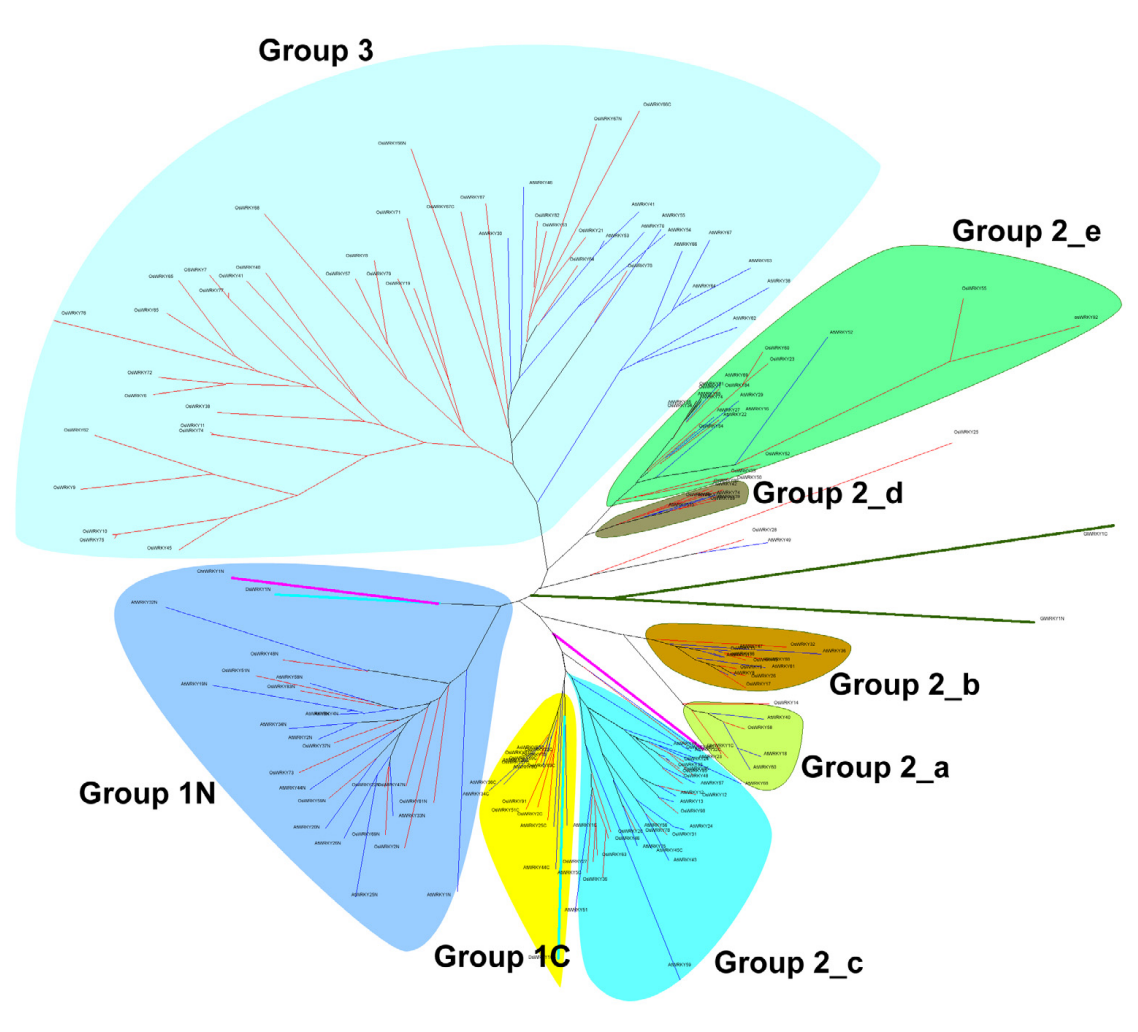
**Unrooted phylogenetic tree of the WRKY domains**. The tree was reconstructed from the amino acid sequences using the neighbor-joining program from Phylip 3.57. Clades of WRKY domains are labelled according to the classifications of AtWRKY domains by Eulgem et al [3] who proposed three groups and five subgroups in Group 2 (a, b, c, d and e). We suggest classifying WRKY domains into five groups modified from the old system. While Groups 1 and 3 are unchanged, the original subgroup 2_c is promoted to Group 2_c. Subgroups 2_a and 2_b, and subgroups 2_d and 2_e are combined to form two new groups, 2_a + 2_b, and 2_d + 2_e, respectively (see text for details). WRKY domains from *G. lamblia *are represented by thick and dark-green branches; the slime mold, thick and cyan; the green alga, thick and magenta; Arabidopsis, thin and blue; and rice, thin and red.

To examine the evolutionary relationships among the WRKY domains, we estimated the phylogeny by using the neighbor-joining program from PHYLIP 3.57 for the amino acid sequences of WRKY domains from *G. lamblia*, the slime mold, the green alga, Arabidopsis and rice. The phylogenetic relationships were also inferred with the programs of the least squares and parsimony from PAUP 4.0 for the corresponding nucleotide sequences. We also did the same analysis for the rice dataset alone. The topology of trees obtained from these analyses is essentially the same, and the neighbor-joining tree is shown in Figure 1. Group 2 domains designated by Eulgem et al. [3] are not monophyletic, but form three distinct clades. These include: 2_a + 2_b, 2_c, and 2_d + 2_e. Moreover, Group 2_a + 2_b and Group 2_c are closely related to Group 1C domains, while Group 3 is clustered with Group 2_d + 2_e. In addition, the rice and Arabidopsis WRKY trees (not shown) consistently clustered WRKY1N domains as a monophyletic subtree and all other domains as a natural clade, supporting the suggestion that Groups 2 and 3 domains are more closely related to the C-terminal domains of Group 1 genes than to the N-terminal domains [3].

**Figure 2:**
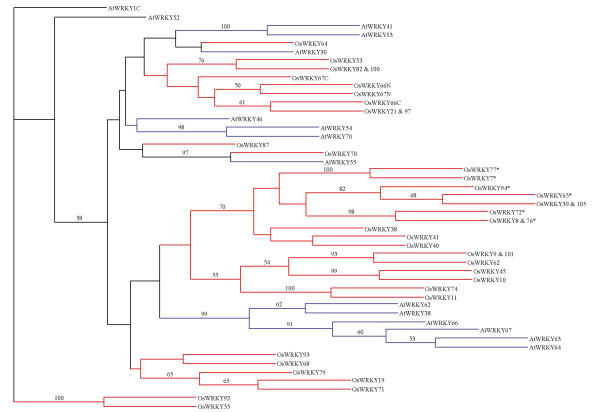
**Phylogram of Group 3 WRKY domains from Arabidopsis (AtWRKY) and rice (OsWRKY)**. The amino acid sequences were analysed with the neighbor-joining and parsimony algorithms implemented in PHYLIP 3.57. Bootstrap values ≥ 50% are indicated above the nodes for distance analysis. The C-terminal domains, AtWRKY1C, was used as the outgroup. OsWRKY proteins with the variant WRKYGEK are marked by *.

In flowering plants, genes encoding WRKY domains appear to have been duplicated independently in monocots and dicots. For Group 3 domains, three subsets each of which consists of five or more members only from rice can be distinguished from the phylogram shown in Figure 2. Similarly, six members of WRKY domains, all from Arabidopsis, are clustered together. Independent domain clusters of either species are also found in other WRKY subfamilies (data not shown). These results suggest that numerous duplications and diversifications for WRKY genes, particularly Group 3 genes, have occurred after the divergence of the monocots and dicots. Indeed, all rice WRKY domains with the sequence WRKYGEK (Table 1) are classified as a sub-cluster of the largest rice domain cluster in Group 3 (Figure 2), implying that multiple duplication events led to this large cluster in rice.

## Discussion

WRKY genes seem to be an innovation in eukaryota after the divergence of eubacteria – archaea – eukaryota. In eukaryotes, the WRKY genes are present in the green plants as well as in the ancient eukaryote *G. lamblia *and the mycetozoan *D. discoideum*, but not in fungi and animals. *G. lamblia *is a primitive unicellular eukaryote diverged ~ 1,500 million years ago (mya) [51]. Originally thought as plant-specific [2,3], the WRKY transcription factors therefore seem to have an early origin in eukaryotes. As the mycetozoa is closely related to the fungi-animal clade [41,43], the WRKY gene(s) may have been lost prior to the divergence of fungi and animals, but after the split of the slime mold and fungi-animal lineages.

**Figure 3:**
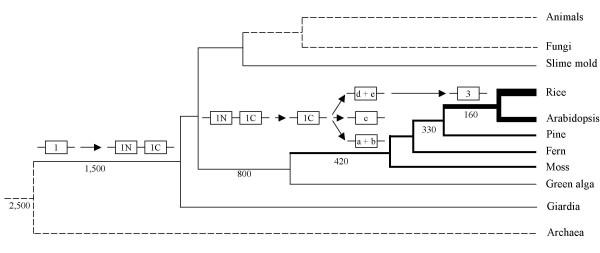
**Model of the origin and duplications of WRKY gene family**. The phylogenetic tree of eukaryotes using the archaea as the outgroup is modified from Baldauf and Doolittle [43] and Kenrick and Crane [50]. The solid lines correspond to branches where WRKY homologues are identified, while the thickness of the line represents the relative size of WRKY family for the branch, from the thinnest for one copy in Giardia, the slime mold and the green alga to the thickest for over 100 copies in rice. The broken lines represent branches where WRKY genes are not present or have not been identified. The WRKY gene is symbolized by the box for the WRKY domain and the lines for sequences around the domain. The text in the box indicates the group the WRKY domain belongs to (1, Group 1; 1N and 1C, N- and C-terminal domains of Group 1 proteins; a + b: Group 2_a + 2_b; c: Group 2_c; d + e, Group 2_d + 2_e; 3: Group 3). The major gene duplications and diversifications are shown above the branch. The number shown below the branch is the divergence time (million years ago) of its children branches. The branch length is not scaled to the evolutionary distance.

Based on the current data, we propose a model for the origin and evolution of the WRKY factor family (Figure 3). First, the ancestor of the descendant WRKY genes found in *G. lamblia*, the slime mold and the green alga seems to be a Group 1 gene encoding two WRKY domains. The conservation of the C- and N-terminal domains suggests that they are derived from a single domain by domain duplication. Therefore we hypothesize that the earliest WRKY factor had one WRKY domain and the gene was innovated post the first appearance of eukaryotes ~ 2,500 mya [52] but prior to the divergence leading to *Giardia *protist, ~ 1,500 mya. Second, our data and the previous results by Eulgem et al. [3] suggest that the WRKY domains of groups 2_a + 2_b, 2_c, 2_d + 2_e and 3 are evolutionarily close to the WRKY1C domain. It seems that Group 1 genes which contain only the C-terminal WRKY domain are ancestors of the descendant WRKY genes in other groups. The N-terminal domain in Group 1 genes may have been lost prior to the gene duplication. As the green alga may have only one WRKY gene which belongs to Group 1, the duplications and diversifications leading to other groups in plants probably occurred some time after the divergence of chlorophytes and streptophytes, ~ 800 mya [53]. Third, the domain structure conservation [see Additional file 2] and the phylogenetic analysis (Figure 1) suggest that the three distinct subsets, Groups 2_a + 2_b, 2c, 2_d + 2_e, may be independently evolved from the Group 1 genes which have only the C-terminal domain. In addition, Group 3 genes appear to share a common ancestor with the clade 2_d + 2_e. The identification of 2_c and 2_d + 2_e genes in the moss EST data [see Additional file 4] suggests that the duplications of the genes in these groups predate the diversification of bryophytes, ~ 420 mya [50]. Although the WRKY genes in Group 2_a + 2_b and Group 3 are identified only from flowering plants in the current data, the origin of these genes seems to have occurred prior to the divergence of monocots and dicots, because the characteristic features of the WRKY domains in Group 3 are highly conserved in Arabidopsis and rice. In addition, multiple copies of Group 3 genes may exist in the common ancestor of monocots and dicots, since clusters with nested Arabidopsis and rice sequences are found in the group (Figure 2).

The classification of the WRKY family in Arabidopsis by Eulgem et al. [3] is not completely based on phylogenetic analysis and therefore does not necessarily reflect the evolutionary relationships among the groups. This is even apparent for the tree of AtWRKY genes built by the authors (see their Figure 3). For example, their Group 2 is not monophyletic, but seems to have several ancestors. Obviously it is necessary to implement a new classification scheme for the WRKY family to reflect the evolution of the WRKY domains. Based on phylogenetic analysis (Figure 1), conserved domain structures and intron positions of the WRKY domains [see Additional file 2, B], we suggest a new classification system modified from Eulgem et al. [3]. Instead of three groups and five subgroups under Group 2 in their classifications, genes are reorganized into five independent groups according to the phylogeny of their WRKY domains, i.e., Group 1, Group 2_a + 2_b, Group 2_c, Group 2_d + 2_e, and Group 3. The relationship between the modified system and the original of Eulgem et al. [3] is as follows. Groups 1 and 3 are unchanged, while Group 2_c corresponds to the subgroup c of the old Group 2. The original subgroups a and b, and d and e in the old Group 2 are combined to become two new groups, 2_a + 2_b, and 2_d + 2_e, respectively.

Our evolutionary analysis of WRKY transcription factors in this study may be important to the understanding of the overall mechanisms of biodiversity in the plant kingdom and the particular functions WRKY genes play in plant regulatory networks. First, the comparative analysis of WRKY factors in lower and higher plants indicates that the WRKY family expands as plants evolve from simpler, unicellular to more complex, multicellular forms. Since WRKY genes seem to play important regulatory roles in plants under abiotic and biotic stresses, and flowering plants which have the largest WRKY family are dominant over non-flowering plants in their distribution on the earth, WRKY genes might be essential for much of the enhanced adaptability of flowering plants to the environment. In comparison with pine, fern and moss, WRKY ESTs of flowering plants seem to be over-represented [see Additional file 4], suggesting that the normal functions of flowering plants might depend to a greater extent on the regulatory roles of these transcription factors. It would be interesting to analyze the functions of genes in Group 3, a greatly amplified group in monocots which are most advanced in evolution and most successful in adaptability. Second, the pairs of Arabidopsis WRKY genes, AtWRKY3 and 4, 8 and 28, 11 and 17, 14 and 35, 18 and 60, 24 and 56, and 38 and 62 share similar expression patterns in response to pathogen inoculation and salicylic acid treatment [23]. Phylogenetic analysis indicates that these pairs of genes are clustered together with high bootstrap value support (data not shown). Thus, the newly duplicated WRKY genes may overlap in functions to better protect the cell or organism from deleterious effects caused by gene mutation or deletion. Moreover, a number of WRKY genes from different phylogenetic groups may be activated by the same physiological or environmental stimulus, such as bacterial pathogen attack [6,25,27,54], viral pathogen attack [23], wounding [30], or senescence [33-35]. The WRKY genes are possibly involved in multiple pathways leading to an array of physiological responses. Nevertheless, the elucidation of the evolution and duplicative expansion of the WRKY genes should provide valuable information on their functions.

## Conclusions

Originally believed to be plant-specific, WRKY transcription factor family has an early origin in eukaryotes and is also present in a slime mold which is more closely related to the lineage of fungi-animals than to plants. WRKY genes have been duplicated many times during evolution in plants, resulting in a large gene family for WRKY proteins in flowering plants. The elucidation of the evolutionary pathway of WRKY family and a new classification system we proposed based on phylogenetic analysis, conserved WRKY domain structures and intron positions should assist the functional characterization of WRKY genes.

## Methods

### Datasets

The annotated genome sequences of rice (*Oryza sativa *spp. *japonica*) (OSA1, released on 7/27/2003) and Arabidopsis (ATH1, released on 4/17/2003) were downloaded from TIGR [55]. OSA1 and ATH1 include nucleotide sequences of genes, mRNA and coding regions, peptide sequences, and the gene structure information such as the start and end of the exons in a gene. For the green alga *Chlamydomonas reinhardtii*, the genome sequence release 1.0 on 2/4/2003 was used [56]. We also downloaded *Giardia lamblia *genome sequence released on 1/1/2003 [57]. GenBank's Non-Redundant (nr), dbEST and taxonomy datasets were downloaded from National Center for Biotechnology Information (NCBI) [58]. TIGR's Gene Indices for plant species [see Additional file 4] and the slime mold *Dictyostelium discoideum *were downloaded from TIGR [59]. These Gene Indices represent non-redundant gene transcripts assembled from publicly available ESTs and annotated sequences [49]. Pfam's WRKY domain sequences (WRKY-seed) were also downloaded [60].

### WRKY gene identification

We searched 'nr' and dbEST datasets for WRKY genes in species outside the plant phyla. The dbEST dataset was also used to survey the expressed WRKY genes in plant species. We aligned the sequences in the datasets with WRKY-seed using BLAST programs [61]. To determine the taxonomical distribution of WRKY genes from the BLAST output, we constructed a database where the BLAST results, the subject sequences and their associated taxonomy information from NCBI [58] were stored. The significant hits (E < 10^-4^) were parsed and manually checked for the presence of the characteristic features of the WRKY domain.

To systemically catalog the WRKY genes for rice and *G. lamblia*, we searched their genome sequences with blastp and PSI-BLAST [61] using WRKY-seed as the query. For PSI-BLAST, we used the default settings for three iterations. We also searched for WRKY genes with HMMER using the global profile of the WRKY domain [60]. HMMER, a sequence analysis tool based on profile Hidden Markov models [62], is available at [63]. The search results with the threshold of E < 10^-4 ^for blastp and PSI-BLAST and E < 0.1 for HMMER were manually compared to remove non-WRKY hits. We also used the same strategy to identify the set of WRKY genes from the Arabidopsis genome.

To identify WRKY genes from the green alga, we first BLASTed its genome sequence against the WRKY-seed. The significantly aligned sequences (E < 10^-4^) were then subject to WRKY domain and gene predictions. The WRKY domain was predicted with the Pfam's DNA SEARCH [64], a web-interface backed by the GeneWise algorithm [65]. The WRKY gene was predicted by FGENESH using the profile for monocots [66,67] and GENSCAN using the profile for maize [68,69].

We also searched ESTs and EST-assembled contigs for the identified WRKY genes of rice, the green alga, *G. lamblia *and the slime mold, using blastn. An EST- or contig-hit was accepted if the identity of the alignment was > 96% for > 400 aligned nucleotides (nt), > 97% for 300 ~ 399 nt, > 98% for 200 ~ 299 nt, > 99% for 100 ~ 199 nt, and = 100% for 50 ~ 99 nt. The alignment with < 50 nt was discarded.

### Analysis of WRKY genes

The WRKY domain boundary was defined as by Eulgem et al. [3]. The peptide sequences of the domains were aligned with ClustalX (v1.81, with default settings) [70] and the alignment was adjusted based on the conserved features of the WRKY domains. The results were then used to guide the alignment of the corresponding nucleotide sequences. The neighbor-joining algorithm implemented in PHYLIP 3.573c [71] for amino acid sequences with the pairwise distance computed under the PAM model, and the least square fit and most parsimony algorithms in PAUP* 4.0b10 [72] for nucleotide sequences were used for phylogenetic tree reconstruction.

## Authors' contributions

LW initiated the study. YZ and LW carried out the analyses, and YZ drafted the manuscript.

## Supplementary Material

Additional File 1 WRKY genes from *Giardia lamblia*, *Dictyostelium discoideum *and *Chlamydomonas reinhadrtii* Click here for file

Additional File 2 Multiple alignments, domain classification and sequence conservation patterns of WRKY domains from rice (OsWRKY), Arabidopsis (AtWRKY), the green alga (ChrWRKY), the slime mold (DsWRKY) and Giardia lamblia (GlWRKY) Click here for file

Additional File 3 Identified members of the WRKY superfamily in the rice genome Click here for file

Additional File 4 Survey of WRKY genes from ESTs or their assembled gene indices for 19 plants and the phylogenetic classification of the genes Click here for file
