# Supplementary material for: The WRKY transcription factor superfamily: its origin in eukaryotes and expansion in plants
Source: BMC Evol Biol. 2005 Jan 3;5:1. doi: 10.1186/1471-2148-5-1 (PMC544883; doi:10.1186/1471-2148-5-1)
Supplement: Additional File 1 — WRKY genes from Giardia lamblia, Dictyostelium discoideum and Chlamydomonas reinhadrtii [file 1471-2148-5-1-S1.pdf]

GlWRKY1, gi|29249388|gb|EAA40901.1|, [*Giardia lamblia*]
MKEGSLHDLGEPIELSVVSQYADKHEPLPGAIGAGGHQNASGVHHQMDVSVHTLTSISADLYNHNSFGRE
RINPVPTLSYGERLPAASSVSDKYLRQITGFDASQAMDLPALSGNLPAVSYLPSSTLPCLPLSAANMGVS
QIVERAAFIAPSNLQANSDLGQNYSNGISVQSIPHIPISGLLPTKAHDFPRDGTLSPASSDDSGIGSSSF
LCSMDQSSCPITSTSSAFQSSRSQSVSLAAPGPIPPSTPSLISRSFSGASRPNISAKDLQTSVSLSSQTR
SATKSFTLLGDSLGGAPSQDGSSTLVASATVSEPRSLRKATTRRVECSIRKTTTERVTIRELPADGYCWR
KYGSKRLPNNSHPKSYFRCSVPGCQAKRYVTETDNRVLKTEYIGEHNHGKSSSTTHEATSLADVCRLAAL
GQNFLIKSDLSLSVLSKTFLDLAVLLRPSIFNNDYLILNGFGSIVLDDSDKRYRDPINGHEIIEISDSIY
PLSALTSMVMTFFAKGVELSNAVNLAITQLNKSFREKAMYAIRKQDSRGRNHSDDSSLVAIPGEDPSTKG
SSAHAPRGRKLIISCKLPSYDQIESSIDFFRWKKYGHKPQTDTRLDSKSYYRCAFFNCPARR TITFFYSL
SSDGTETVESVIVQYENQHTHPPDRTRIPYKDCLKLLKAPDHLPIQPIRSPDHPIFANPTSYLGVTGVTV
AQRPVFTSLTPHMTAPIGDPAGHHPIAMQHGMPAQASHGISDINTVTHGAQLVSSMQRSDSFERTSCLLF
PRVDSMALQEGLSGLHTDQSLYGCASGSPGYGASYPLATQPDCSSYSLVRSNSFLFLNQLGQAQSQLDQV
IDAGHSSTVGSFGSTSKIPSITPHSIHSSNMEKDLLSYCSTIVRGDENINLTNAENESISRMHDTLLGPM
VNVYS

DsWRKY1, gi|28829829|gb|AAO52331.1|, [*Dictyostelium discoideum*]
MGAQYSTELNKYNNNNNNNNNNNNNNNNNNNHTISGNENLNNNNNNNNNNNNNYNNNNNNNNNNNNNNNN
NNNNNNNNNNNNNNNNKNNGNSNSNSNNNNNNNGINKYSNYFESLINNTNKDTNIPNNSNSNNNNSDRDF
IQNMIKSANQIKPGEKELVLFHKLHQSLKENQSFSIEELRNSSILSISPDKSFLTNINEINVNNNNNNNN
NNNENNKVGVNGSSTTTTTTTTTTTNNNSNNNNNNNNNNNNNNNNNNNNNEDDEDDYGDDDTIENNEPTF
VDKPNKSAKLQKPKLKSNLNDTNGGNSPQQNGKISKYMAKKLLALQQKQLEQEQEQKQQQKQQQQQQQQQ
QQQQQQQQQQQQKDAIENINNNNNNKLQPIVKNSVNKTTKQKKNKTTTHNVKQQITFLTKSDNEDEYDAS
DEYIDDDDDDDEKYDDDDDEYFEGNNNNNYKKNNITNKSKNKNNNDESDSFSESEIFKQKKLNHDKNQSN
PKQQLTSHSEFDNSLLNKNQSRTNSKIQKLQIKEENYHQIQQEHGEKQQQQQQQQQQPQQQQQQQQQQQQ
QQQQEMQVDKEQTEKIINTNKKEEQKPNDYFPPIPTNPFKRDNETAFGKNNENNNNNNNNNNNNNNNNNN
NNNNNNNNYRNNKPNGEGFLSNLKIIPPMISSSPYYSKKSSNVVPTSPKSNLSDQQPPFSPVQISPQKQS
PATTTTTTTTTTPTPLKKQKKNKTNNNINNNNINNNNNNNNNNNNNNNNNNNNNNNNNNNYSINNINNEED
EENNSTQNNNNNNNNNNNSPQNSNTNSNNSNNSNNSNNISNIVSDGYQWRKYGQKNVKGSLHPRHYYKCT
FQGCNVRKQVERIGDTNQNSTVYKGEHCHGFPQTTRVVSDQQAFRNSVMFEGLDGNNNNNNNNNNNNNNY
SSNSNSNGNGNGNGNGNGNGNGNGNSNGNQDQNGNSFNDQNGDSPTQHGQISPMNSPKNTIPTTTTTT
TSISTYVNTNSTNKKNNSKQEKKISVKNETTDDDEFQEDIDQLSNNNNNNNNNNNNNNNNNNNNNNNNNN
NNNNNNNNNNNNNNNNNNDDNNNNNNNNNNNNNNNRFNGTSESKGSKKLVIETGSSIDHLDDGFFWRKYG
QKSVKGSPFPKSYFKCAELTCPVKKQVIQQDSKYINTYRGKHNHDPPESEAIEKRKKHFNGLYNNNNNNN
NNNNNNNNNNNNNNNINNINNNNINNLNSLNNINNINNNNNSNQINNFSGSIQNNLKNILKEQLKEAGKL
HNEIENNLIDD

ChrWRKY1, predicted by GENSCAN from scaffold_1387, [*Chlamydomonas reinhadrtii*]
XFKRGSDWAPRTTRLFCFHYSRFVRSGRCGRVAEQGENSPSALTVRQGVKVAPLTTPQQVEASPTTGIHQ
LCPPLFQPAQPARVPLPIPARTEAASAAPEPTRAIKREYEPRAGNGKQSVANSDGWQWRKYGEKLVKGSP
NPRSYYKCSHPGCLAKKIVERSDSDGTVLSTEYKGDHCHPAPSAVKASRFKPKPKTEPPVMVAPPVFSAV
DITVPNGFPPGANGRVGFPLSGGDMLPIPEALKSDFPVPHAAGAAAAHEDDTDTSEPEPAAALKAAPQDT
RAAQAAATAIRKVRDSAESPSKRLDMLAAYAEEAERQLKSSSNSPEQGPSAKRQRTEAGAMRTRANPDDD
DDGSGAPSTSGMQRVVDITNMDDGYRWRKYGQKQVKGSPFPRAYYKCTHMGCSVRKHVERSAEDETRFVV
TYEGTHSHRLPTGSRRRSARDMAEDDEDYEGEDAEEDSSQPTSPQYGNVNGSGGPGQHAASKAAAQGAQL
VHPSGAQPASADFGQQLQQLSTSLLASTVLQQAALSGVLPLLQYNSLSSEALASLGVNSEALQGVEQLNL
ASVGNLADLTNLLRQHAQMDLALAAQAQAIDAANANWDPLACLITPRPNVSPAGQGHAMGQAPSAGTGRQ
TKAAVFQKQVATTEA

Additional File 1. WRKY genes from *Giardia lamblia*, *Dictyostelium discoideum* and *Chlamydomonas reinhadrtii*. WRKY domains are highlighted and the WRKYGQK or its variants and the zinc-finger motif are in colors.
