# Supplementary material for: The WRKY transcription factor superfamily: its origin in eukaryotes and expansion in plants
Source: BMC Evol Biol. 2005 Jan 3;5:1. doi: 10.1186/1471-2148-5-1 (PMC544883; doi:10.1186/1471-2148-5-1)
Supplement: Additional File 2 — Multiple alignments, domain classification and sequence conservation patterns of WRKY domains from rice (OsWRKY), Arabidopsis (AtWRKY), the green alga (ChrWRKY), the slime mold (DsWRKY) and Giardia lamblia (GlWRKY) [file 1471-2148-5-1-S2.pdf]

A

GROUP 1N

```
OsWRKY15    DTHPPEYDGYNWRICGQKVVQGGCHQKFYYECSQANCGAEKSVTRSA-DGQIKKTVCKGSHNHPLS
OsWRKY16    YPNPTEYDGYNWRMCGQKLVQGGCHQKFYYECSQANCGAEKSVTRSA-DGQIKKTVCKGSHNHPRS
OsWRKY2N    AIEQPAKDGYNWRKYGQKQLKDAESPRSYYKCTRDGCPVKKIVERSS-DGCIKEITYKGRHSHPRP
OsWRKY47N   LIITPAEDGYNWRKYGQKQVKNSEHPRSYYKCTFTNCAVKK-VERSQ-DGQITEIVYKGSHNHPLP
OsWRKY51N   VLDRPTDDGYNWRKYGQKAVKGGEYPKSYYKCTHLNCLVRKNVEHSA-DGRIVQIIYRGQHTHERP
OsWRKY103N  VLDRPTDDGYNWRKYGQKAVKGGEYPKSYYKCTHLNCLVRKNVEHSA-DGRIVQIIYRGQHTHERP
AtWRKY58N   NVDKPADDGYNWRKYGQKPIKGCEYPRSYYKCTHVNCPVKKKVERSS-DGQITQIIYKGQHDHERP
OsWRKY83N   PVDKPADDGYNWRKYGQKVVKGSDCPRSYYKCTHPNCPVKKKVEHAE-DGQISEIIYKGKHNHQRP
AtWRKY3N    NADKPADDGYNWRKYGQKQVKGSDFPRSYYKCTHPACPVKKKVERSL-DGQVTEIIYKGQHNHELP
AtWRKY4N    NVDKPADDGYNWRKYGQKQVKGSEFPRSYYKCTNPGCPVKKKVERSL-DGQVTEIIYKGQHNHEPP
AtWRKY19N   NVDKQVNDGYNWQKYGQKKVKGSKFPLSYYKCTYLGCPSKRKVERSL-DGQVAEIVYKDRHNHEPP
OsWRKY22N   SQRRSSDDGYNWRKYGQKQVKGSENPRSYYKCTFPNCPTKKKVERSL-DGQITEIVYKGTHNHAKP
OsWRKY69N   QSRRSSDDGYNWRKYGQKQMKGSENPRSYYKCTFPGCPTKKKVEQSP-DGQVTEIVYKGAHSHPKP
AtWRKY33N   REQRKGEDGYNWRKYGQKQVKGSENPRSYYKCTFPNCPTKKKVERSL-EGQITEIVYKGSHNHPKP
AtWRKY26N   SSNKTSDDGYNWRKYGQKQVKGSENPRSYFKCTYPNCLTKKKVETSLVKGQMIEIVYKGSHNHPKP
AtWRKY25N   MVSRNSNDGYGWRKYGQKQVKKSENPRSYFKCTYPDCVSKKIVETAS-DGQITEIIYKGGHNHPKP
OsWRKY61N   GGNNKLEDGYNWRKYGQKQVKGSENPRSYYKCTYNGCSMKKKVERSLADGRITQIVYKGAHNHPKP
OsWRKY73    AKNSLSYDGYSWRKYGQKQVKGSEFPRSYYKCTHPTCPVKRKVEMTP-DGRIAEIVYNGEHNHPKP
OsWRKY104   AKNSLSYDGYSWRKYGQKQVKGSEFPRSYYKCTHPTCPVKRKVEMTP-DGRIAEIVYNGEHNHPKP
AtWRKY44N   TGDRSSVDGYNWRKYGQKQVKGSECPRSYYKCTHPKCPVKKKVERSV-EGQVSEIVYQGEHNHSKP
AtWRKY2N    AGGAPAEDGYNWRKYGQKLVKGSEYPRSYYKCTNPNCQVKKKVERSR-EGHITEIIYKGAHNHLKP
AtWRKY34N   ACCAPADDGYNWRKYGQKLVKGSEYPRSYYKCTHPNCEAKKKVERSR-EGHIIEIIYTGDHIHSKP
OsWRKY37N   AAAAVAEDGYSWRKYGQKQVKHSEYPRSYYKCTHASCAVKKKVERSH-EGHVTEIIYKGTHNHPKP
OsWRKY59N   VAEKSAEDGYNWRKYGQKHVKGSENPRSYYKCTHPNCDVKKLLERSL-DGQITEVVYKGRHNHPKP
AtWRKY20N   TPSILADDGYNWRKYGQKHVKGSEFPRSYYKCTHPNCEVKKLFERSH-DGQITDIIYKGTHDHPKP
AtWRKY1N    IREKVMEDGYNWRKYGQKLVKGNEFVRSYYRCTHPNCKAKKQLERSA-GGQVVDTVYFGEHDHPKP
OsWRKY48N   VSVNMVGDGFNWRKYGQKQVKSSENSRSYYRCTNSNCLAKKKVEHCPD-GRVVEIIYRGTHNHEPP
AtWRKY32N   VPRTPARDGYNWRKYGQKQVKSPKGSRSYYRCTYTECCAKK-IECSNDSGNVVEIVNKGLHTHEPP
DsWRKY1N    NISNIVSDGYQWRKYGQKNVKGSLHPRHYYKCTFQGCNVRKQVER-IGDTNQNSTVYKGEHCHGFP
ChrWRKY1N   KQSVANSDGWQWRKYGEKLVKGSPNPRSYYKCSHPGCLAKKIVERSDSDGTVLSTEYKGDHCHPAP
GlWRKY1N    TIRELPADGYCWRKYGSKRLPNNSHPKSYFRCSVPGCQAKRYVTETD--NRVLKTEYIGEHNHGKS
```

GROUP 1C

**OsWRKY2C**	SEVDLLDDGYRWRKYGQK-VVKGNPRPRSYYKCTADGCNVRK-------QIERASADPKCVLTTYTGRHNHDPP
**OsWRKY83C**	SEVDLLDDGYRWRKYGQK-VVKGNPHPRSYYKCTYAGCNVRK-------HIERASSDPKAVITTYEGKHNHEPP
**OsWRKY51C**	SEIDLLDDGYRWRKYGQK-VVKGNPYPRSYYKCTYLGCDVKK-------QVERSVEEPNAVITTYEGKHIHDVP
**OsWRKY103C**	SEIDLLDDGYRWRKYGQK-VVKGNPYPRSYYKCTYLGCDVKK-------QVERSVEEPNAVITTYEGKHIHDVP
**AtWRKY3C**	SEVDLLDDGYRWRKYGQK-VVKGNPYPRSYYKCTTPDCGVRK-------HVERAATDPKAVVTTYEGKHNHDVP
**AtWRKY4C**	SEVDLLDDGYRWRKYGQK-VVKGNPYPRSYYKCTTPGCGVRK-------HVERAATDPKAVVTTYEGKHNHDLP
**AtWRKY58C**	SEVDLLDDGYRWRKYGQK-VVKGNPHPRSYYKCTTPNCTVRK-------HVERASTDAKAVITTYEGKHNHDVP
**AtWRKY26C**	SDIDILDDGYRWRKYGQK-VVKGNPNPRSYYKCTFTGCFVRK-------HVERAFQDPKSVITTYEGKHKHQIP
**AtWRKY34C**	SDIDILDDGYRWRKYGQK-VVKGNPNPRSYYKCTANGCTVTK-------HVERASDDFKSVLTTYIGKHTHVVP
**OsWRKY13**	SEVDILDDGYRWRKYGQK-VVKGNPNPRSYYKCTHQGCSVRK-------HVERASHDLKSVITTYEGKHNHEVP
**OsWRKY47C**	SEVDILDDGYRWRKYGQK-VVKGNPNPRSYYKCTHPGCSVRK-------HVERSSHDLKSVITTYEGKHNHEVP
**OsWRKY37C**	SEVDILDDGYRWRKYGQK-VVKGNPNPRSYYKCTHPGCLVRK-------HVERASHDLKSVITTYEGKHNHEVP
**OsWRKY59C**	SEVDILDDGYRWRKYGQK-VVKGNPNPRSYYKCTNTGCPVRK-------HVERASHDPKSVITTYEGKHNHEVP
**AtWRKY2C**	SDVDILDDGYRWRKYGQK-VVKGNPNPRSYYKCTAPGCTVRK-------HVERASHDLKSVITTYEGKHNHDVP
**AtWRKY20C**	SEVDILDDGYRWRKYGQK-VVRGNPNPRSYYKCTAHGCPVRK-------HVERASHDPKAVITTYEGKHDHDVP
**OsWRKY22C**	SDIDILDDGYRWRKYGQK-VVKGNPNPRSYYKCTTAGCPVRK-------HVERASHDLRAVITTYEGKHNHDVP
**OsWRKY69C**	SDIDILDDGYRWRKYGQK-VVKGNPNPRSYYKCTTAGCPVRK-------HVERASNDLRAVITTYEGKHNHDVP
**OsWRKY61C**	SDIDILDDGFRWRKYGQK-VVKGNPNPRSYYKCTTVGCPVRK-------HVERASHDTRAVITTYEGKHNHDVP
**AtWRKY33C**	SDIDILDDGYRWRKYGQK-VVKGNPNPRSYYKCTTIGCPVRK-------HVERASHDMRAVITTYEGKHNHDVP
**AtWRKY10C**	SDEDNPNDGYRWRKYGQK-VVKGNPNPRSYFKCTNIECRVKK-------HVERGADNIKLVVTTYDGIHNHPSP
**AtWRKY25C**	SDIDVLIDGFRWRKYGQK-VVKGNTNPRSYYKCTFQGCGVKK-------QVERSAADERAVLTTYEGRHNHDIP
**AtWRKY45C**	SQVDILDDGYRWRKYGQK-AVKNNPFPRSYYKCTEEGCRVKK-------QVQRQWGDEGVVVTTYQGVHTHAVD
**AtWRKY44C**	VESDSLEDGFRWRKYGQK-VVGGNAYPRSYYRCTSANCRARK-------HVERASDDPRAFITTYEGKHNHHLL
**AtWRKY1C**	TLFDIVNDGYRWRKYGQK-SVKGSPYPRSYYRCSSPGCPVKK-------HVERSSHDTKLLITTYEGKHDHDMP
**ChrWRKY1C**	DITN-MDDGYRWRKYGQK-QVKGSPFPRAYYKCTHMGCSVRK-------HVERSAEDETRFVVTYEGTHSHRLP
**DsWRKY1C**	NISNIVSDGYQWRKYGQK-NVKGSLHPRHYYKCTFQGCNVRK-------QVER-IGDTNQNSTVYKGEHCHGFP
**AtWRKY32C**	GDVGICGDGYRWRKYGQK-MVKGNPHPRNYYRCTSAGCPVRK-------HIETAVENTKAVIITYKGVHNHDMP
**OsWRKY48C**	VQAGKTSDGYRWRKYGQK-IVKGNPNPRSYYRCTHDGCPVRK-------HVEKAPDDDNNIVVTYEGKHNHDQP
**GlWRKY1C**	DQIESSIDFFRWKKYGHKPQTDTRLDSKSYYRCAFFNCPARRTITFFYSLSSDGTETVESVIVQYENQHTHPPD

GROUP 2_a + 2_b

Domains inside and above the box correspond to subfamily 2a and 2b in the classification by Eulgem et al. (2000), respectively.

```
OsWRKY86   SDAPMISDGCQWRKYGQKMAKGNPCPRAYYRCTMAAGCPVRKQVQRCAED-RTVLITTYEGNHNHPLP
AtWRKY42   SEAPMLSDGCQWRKYGQKMAKGNPCPRAYYRCTMAVGCPVRKQVQRCAED-RTILITTYEGNHNHPLP
OsWRKY29   SEAPIIADGCQWRKYGQKMAKGNPCPRAYYRCTMATGCPVRKQVQRCAED-RSILITTYEGTHNHPLP
OsWRKY96   SEAPIIADGCQWRKYGQKMAKGNPCPRAYYRCTMATGCPVRKQVQRCAED-RSILITTYEGTHNHPLP
AtWRKY6    SEAPMISDGCQWRKYGQKMAKGNPCPRAYYRCTMATGCPVRKQVQRCAED-RSILITTYEGNHNHPLP
AtWRKY31   SEAAMISDGCQWRKYGQKMAKGNPCPRAYYRCTMAGGCPVRKQVQRCAED-RSILITTYEGNHNHPLP
OsWRKY43   SEAPMISDGCQWRKYGQKMAKGNPCPRAYYRCTMASQCPVRKQVQRCAKD-KSILITTYEGTHSHPLP
OsWRKY88   SEAPMISDGCQWRKYGQKMAKGNPCPRAYYRCTMAIGCPVRKQVT------KLIYM------HSHN—
AtWRKY47   SDATTVNDGCQWRKYGQKMAKGNPCPRAYYRCTMAVGCPVRKQVQRCAED-TTILTTTYEGNHNHPLP
AtWRKY36   CEDPSINDGCQWRKYGQKTAKTNPLPRAYYRCSMSSNCPVRKQVQRCGEEETSAFMTTYEGNHDHPLP
OsWRKY26   CDTPTMNDGCQWRKYGQKISKGNPCPRAYYRCTVAPNCPVRKQVQRCADD-MSILITTYEGTHSHPLP
AtWRKY72   CDTPTMNDGCQWRKYGQKIAKGNPCPRAYYRCTVAPGCPVRKQVQRCADD-MSILITTYEGTHSHSLP
OsWRKY5    CDAPTMNDGCQWRKYGQKIAKGNPCPRAYYRCTVAAGCPVRKQVQRCADD-MSILITTYEGTHNHPLS
OsWRKY17   CQGPTMNDGCQWRKYGQKVAKGNPCPRAYYRCTVAPGCPVRKQVQRCLED-MSILVTTYEGTHNHPLP
AtWRKY9    CETATMNDGCQWRKYGQKTAKGNPCPRAYYRCTVAPGCPVRKQVQRCLED-MSILITTYEGTHNHPLP
AtWRKY61   KTRVSMNDGCQWRKYGQKIAKGNPCPRAYYRCTIAASCPVRKQVQRCSED-MSILISTYEGTHNHPLP
OsWRKY32   CSAPTVKDGCQWRKYGQKTAKGNPWPRGYYRCTGAPGCPVKKQVQRCNHD-TSVLVTTYDGVHNHPIT
OsWRKY4    DLSLVVKDGYQWRKYGQKVTKDNPCPRAYFRCSFAPACPVKKKVQRSADD-NTVLVATYEGEHNHAQP
OsWRKY18   DLSLVVKDGYQWRKYGQKVTKDNPCPRAYFRCSFAPACPVKKKVQRSAED-NTILVATYEGEHNHGQP
OsWRKY58   DTSLVVKDGYQWRKYGQKVTRDNPSPRAYFRCAFAPSCPVKKKVQRSAED-SSLLVATYEGEHNHPHP
AtWRKY40   DTTLVVKDGYQWRKYGQKVTRDNPSPRAYFKCACAPSCSVKKKVQRSVED-QSVLVATYEGEHNHPMP
AtWRKY18   DTSLTVKDGFQWRKYGQKVTRDNPSPRAYFRCSFAPSCPVKKKVQRSAED-PSLLVATYEGTHNHLGP
AtWRKY60   DTSLTVKDGYQWRKYGQKITRDNPSPRAYFRCSFSPSCLVKKKVQRSAED-PSFLVATYEGTHNHTGP
OsWRKY14   SMAETVKDGYQWRKYGQKVTRDNPYPRAYFRCAFAPSCPVKKKLQRCAED-RSMLVATYEGEHNHALS
```

GROUP 2_c

**OsWRKY28**	NNGGLADDGYKWRKYGQKSIKNSPNPRSYYRCTNPRCNAKKQVERAVDEPDTLIVTYEGLHLHYTY
**AtWRKY49**	NSNGMCDDGYKWRKYGQKSIKNSPNPRSYYKCTNPICNAKKQVERSIDESNTYIITYEGFHFHYTY
**OsWRKY27**	SEVEVLDDGFKWRKYGKKAVKSSPNPRNYYRCSAAGCGVKKRVERDGDDPRYVVTTYDGVHNHATP
**OsWRKY63**	SEVEILDDGFKWRKYGKKAVKNSPNPRNYYRCSTEGCNVKKRVERDREDHRYVITTYDGVHNHASP
**OsWRKY36**	SDDEILDDGYKWRKYGKKSVKNSPNPRNYYRCSTEGCNVKKRVERDKNDPRYVVTMYEGIHNHVCP
**OsWRKY46**	SEIEILDDGYKWRKYGKKSVKNSPNPRNYYRCSTEGCNVKKRVERDKDDPSYVVTTYEGTHNHVSP
**AtWRKY50**	SEVEVLDDGFKWRKYGKKMVKNSPHPRNYYKCSVDGCPVKKRVERDRDDPSFVITTYEGSHNHSSM
**AtWRKY51**	SKIDVMDDGFKWRKYGKKSVKNNINKRNYYKCSSEGCSVKKRVERDGDDAAYVITTYEGVHNHESL
**OsWRKY20**	SEVDVLDDGYRWRKYGKKMVKNSPNPRNYYRCSSEGCRVKKRVERARDDARFVVTTYDGVHNHPAP
**OsWRKY12**	SEVDVLDDGYKWRKYGQKVVKNSLHPRSYFRCTHSNCRVKKRVERLSTDCRMVITTYEGRHTHSPC
**OsWRKY98**	SEVDVLDDGYKWRKYGQKVVKNSLHPRSYFRCTHSNCRVKKRVERLSTDCRMVITTYEGRHTHSPC
**OsWRKY99**	SEVDVLDDGYKWRKYGQKVVKNSLHPRSYFRCTHSNCRVKKRVERLSTDCRMVITTYEGRHTHSPC
**AtWRKY12**	SDVDVLDDGYKWRKYGQKVVKNSLHPRSYYRCTHNNCRVKKRVERLSEDCRMVITTYEGRHNHIPS
**OsWRKY90**	SDVDVLDDGYKWRKYGQKVVKNTQHPRSYYRCTQDNCRVKKRVERLAEDPRMVITTYEGRHVHSPS
**AtWRKY13**	SEVDVLDDGYRWRKYGQKVVKNTQHPRSYYRCTQDKCRVKKRVERLADDPRMVITTYEGRHLHSPS
**AtWRKY24**	SDDDVLDDGYRWRKYGQKSVKHNAHPRSYYRCTYHTCNVKKQVQRLAKDPNVVVTTYEGVHNHPCE
**AtWRKY56**	SDDDVLDDGYRWRKYGQKSVKNNAHPRSYYRCTYHTCNVKKQVQRLAKDPNVVVTTYEGVHNHPCE
**OsWRKY31**	SDNDILDDGYRWRKYGQKAVKNSKHPRSYYRCTHHTCNVKKQVQRLAKDTSIVVTTYEGVHNHPCE
**AtWRKY43**	SDADILDDGYRWRKYGQKSVKNSLYPRSYYRCTQHMCNVKKQVQRLSKETSIVETTYEGIHNHPCE
**OsWRKY78**	SQVDILDDGYRWRKYGQKAVKNNKFPRSYYRCTHQGCNVKKQVQRLSRDETVVVTTYEGTHTHPIE
**AtWRKY75**	SQVDILDDGYRWRKYGQKAVKNNKFPRSYYRCTYGGCNVKKQVQRLTVDQEVVVTTYEGVHSHPIE
**OsWRKY3**	SEIDHLEDGYRWRKYGQKAVKNSPFPRSYYRCTNSKCTVKKRVERSSDDPSVVITTYEGQHCHHTA
**OsWRKY49**	SEIDHLEDGYRWRKYGQKAVKNSPFPRSYYRCTNSKCTVKKRVERSSDDPSVVITTYEGQHSHHTV
**AtWRKY57**	SDVDNLEDGYRWRKYGQKAVKNSPFPRSYYRCTNSRCTVKKRVERSSDDPSIVITTYEGQHCHQTI
**AtWRKY48**	SDIDNLDDGYRWRKYGQKAVKNSPYPRSYYRCTTVGCGVKKRVERSSDDPSIVMTTYEGQHTHPFP
**OsWRKY24**	SEVDHLEDGYRWRKYGQKAVKNSPYPRSYYRCTTPKCGVKKRVERSYQDPSTVITTYEGQHTHHSP
**OsWRKY44**	SEVDHLEDGYRWRKYGQKAVKNSSYPRSYYRCTAPRCGVKKRVERSEQDPSMVITTYEGQHTHPSP
**OsWRKY35**	SEVDHLEDGYRWRKYGQKAVKNSPYPRSYYRCTTQKCPVKKRVERSYQDPAVVITTYEGKHTHPIP
**OsWRKY85**	SEVDHLEDGYRWRKYGQKAVKNSPFPRSYYRCTTQKCPVKKRVERSYQDAAVVITTYEGKHTHPIP
**AtWRKY8**	TEVDHLEDGYRWRKYGQKAVKNSPYPRSYYRCTTQKCNVKKRVERSYQDPTVVITTYESQHNHPIP
**AtWRKY28**	SEVDHLEDGYRWRKYGQKAVKNSPYPRSYYRCTTQKCNVKKRVERSFQDPTVVITTYEGQHNHPIP
**AtWRKY71**	SEIDHLEDGYRWRKYGQKAVKNSPYPRSYYRCTTQKCNVKKRVERSFQDPSIVITTYEGKHNHPIP
**AtWRKY23**	SEVDHLEDGYRWRKYGQKAVKNSPFPRSYYRCTTASCNVKKRVERSFRDPSTVVTTYEGQHTHISP
**AtWRKY68**	SEVLHLDDGYKWRKYGQKPVKDSPFPRNYYRCTTTWCDVKKRVERSFSDPSSVITTYEGQHTHPRP
**AtWRKY59**	DEKVALDDGYKWRKYGKKPITGSPFPRHYHKCSSPDCNVKKKIERDTNNPDYILTTYEGRHNHPSP

GROUP 2_d + 2_e

Domains inside and above the box correspond to subfamily 2d and 2e in the classification by Eulgem et al. (2000), respectively.

```
OsWRKY33   GEGNTPTDSWAWRKYGQKPIKGSPFPRAYYRCSSSKGCPARKQVERSRNDPDTVIVTYSFEHN-HSAT
OsWRKY84   GEGPPPSDSWAWRKYGQKPIKGSPYPRGYYRCSSSKGCPARKQVERSRADPTVLLVTYSFEHN-HPWP
AtWRKY69   GEVYPPSDSWAWRKYGQKPIKGSPYPRGYYRCSSSKGCPARKQVERSRVDPSKLMITYACDHN-HPFP
AtWRKY65   GDTTPPSDSWAWRKYGQKPIKGSPYPRGYYRCSSTKGCPARKQVERSRDDPTMILITYTSEHN-HPWP
OsWRKY34   SGEVVPSDLWAWRKYGQKPIKGSPYPRGYYRCSSSKGCSARKQVERSRTDPNMLVITYTSEHN-HPWP
OsWRKY57   SGEVVPSDLWAWRKYGQKPIKGSPYPRGYYRCSSSKGCSARKQVERSRTDPNMLVITYTSEHN-HPWP
AtWRKY35   SGEVVPSDLWAWRKYGQKPIKGSPYPRGYYRCSSSKGCSARKQVERSRTDPNMLVITYTSEHN-HPWP
AtWRKY14   SGEVVPSDLWAWRKYGQKPIKGSPFPRGYYRCSSSKGCSARKQVERSRTDPNMLVITYTSEHN-HPWP
OsWRKY1    GGEVVPSDLWAWRKYGQKPIKGSPYPRGYYRCSSSKGCSARKQVERSRADPTMLVVTYTSDHN-HPWP
OsWRKY81   GGEVIPSDLWAWRKYGQKPIKGSPYPRGYYRCSSSKGCMARKQVERSRSDPNMLVITYAAEHN-HPWP
OsWRKY23   ADGGVSSDLWAWRKYGQKPIKGSPYPRGYYKCSSMKGCMARKMVERSPAKPGMLVVTYMAEHC-HPVP
OsWRKY60   ADGGVSTDLWAWRKYGQKPIKGSPYPRGYYKCSSLKACMARKMVERSPEKPGVLVITYIAEHC-HAVP
AtWRKY16   DRGSRSSDLWVWRKYGQKPIKSSPYPRSYYRCASSKGCFARKQVERSRTDPNVSVITYISEHN-HPFP
AtWRKY27   TQENLSSDLWAWRKYGQKPIKGSPYPRNYYRCSSSKGCLARKQVERSNLDPNIFIVTYTGEHT-HPRP
AtWRKY29   KEENLLSDAWAWRKYGQKPIKGSPYPRSYYRCSSSKGCLARKQVERNPQNPEKFTITYTNEHN-HELP
AtWRKY22   AAEALNSDVWAWRKYGQKPIKGSPYPRGYYRCSTSKGCLARKQVERNRSDPKMFIVTYTAEHN-HPAP
OsWRKY54   PADGVSADVWAWRKYGQKPIKGSPYPRGYYRCSSSKGCPARKQVERSRSDPNTFILTYTGEHN-HSAP
OsWRKY6    AASGPAPDLWAWRKYGQKPIKGSPYPRGYYRCSSNKNCAARKQVERCRFDPSFLLLTYTGAHSGHDVP
OsWRKY52   VEQGPPADSWSWRKYGQKPIKGSPHPRGYYKCSSYRGCPARKQVDKCRNDASLLIITYTSDHN-H
AtWRKY11   KIADIPPDEYSWRKYGQKPIKGSPHPRGYYKCSTFRGCPARKHVERALDDPAMLIVTYEGEHR-HNQS
AtWRKY17   KIADIPPDEYSWRKYGQKPIKGSPHPRGYYKCSTFRGCPARKHVERALDDSTMLIVTYEGEHR-HHQS
OsWRKY30   KIADIPPDEYSWRKYGQKPIKGSPYPRGYYKCSTVRGCPARKHVERATDDPAMLVVTYEGEHR-HTPG
AtWRKY39   KIADIPPDEYSWRKYGQKPIKGSPHPRGYYKCSSVRGCPARKHVERCIDETSMLIVTYEGEHN-HSRI
AtWRKY74   KIADIPPDEYSWRKYGQKPIKGSPHPRGYYKCSSVRGCPARKHVERCVEETSMLIVTYEGEHN-HSRI
OsWRKY89   KIADIPPDEYSWRKYGQKPIKGSPHPRGYYKCSSVRGCPARKHVERCVDDPAMLIVTYEGEHN-HTRG
AtWRKY7    KMADIPSDEFSWRKYGQKPIKGSPHPRGYYKCSSVRGCPARKHVERALDDAMMLIVTYEGDHN-HALV
AtWRKY15   KMSDVPPDDYSWRKYGQKPIKGSPHPRGYYKCSSVRGCPARKHVERAADDSSMLIVTYEGDHN-HSLS
AtWRKY21   KVADIPPDDYSWRKYGQKPIKGSPYPRGYYKCSSMRGCPARKHVERCLEDPAMLIVTYEAEHN-HPKL
OsWRKY42   KVADIPADDFSWRKYGQKPIKGSPFPRGYYKCSTLRGCPARKHVERDPTDPSMLIVTYEGEHR-HSPS
OsWRKY50   RNADIPADDYSWRKYGQKPIKGSPYPRGYYKCSTVRGCPARKHVERDPGEPAMLIVTYDGDHR-HGEP
OsWRKY56   RVADIPADEYSWRKYGQKPIKGSPYPRGYYRCSTVKGCPARKHVERAADDPATLVVTYEGDHR-HSPP
OsWRKY80   KVADIPSDNYSWRKYGQKPIKGSPHPRGYYRCSSKKDCPARKHVERCRSDPAMLLVTYENEHN-HAQP
OsWRKY102  KVADIPSDNYSWRKYGQKPIKGSPHPRGYYRCSSKKDCPARKHVERCRSDPAMLLVTYENEHN-HAQP
```

GROUP 3

```
OsWRKY7     TPVP-HEDG--FQWRKYGEKKIQGTH-FTR---SYFRCTYRDD-RGCQATKQIQQ----KDKND-----PPMFQVTYSNEHTCTTT
OsWRKY77    TVVP-HEDG--YQWRKYGEKKIQGTH-FTR---SYFRCTYRDD-RGCQATKQIQQ----EDKND-----PPMFQVTYSNEHTCTTT
OsWRKY8     TYAP-YDDG--HQWRKYGEKKLSNSN-FPR---FYYRCTYKND-MKCPATKQVQQ----KDTND-----PPLFSVTYFNHHTCNSS
OsWRKY76    TYAP-YDDG--HQWRKYGEKKLSNSN-FPR---FYYRCTYKND-MKCPATKQVQQ----KDTND-----PPLFSVTYFNHHTCNSS
OsWRKY72    TFAP-HNDG--HQWRKYGEKKINNCN-FPR---YYYRCTYKDN-MNCPATKQIQQ----KDYSD-----PPLYSVTYYNEHTCNSA
OsWRKY39    TFTP-HFDG--HLWRKYGQKNIKDSA-FPR---LYYRCSYRED-RQCLASKLVQQ----ENDDD-----PPLYRVTYTYEHTCNTT
OsWRKY105   TFTP-HFDG--HLWRKYGQKNIKDSA-FPR---LYYRCSYRED-RQCLASKLVQQ----ENDDD-----PPLYRVTYTYEHTCNTT
OsWRKY65    TPIP-HTDG--HLWRKYGEKKIKNSS-FPR---LYYRCSYRDD-RNCMATKVVQQ----ENDAD-----PPLYRVTYIHPHTCNP-
OsWRKY94    TTTP-ENDG--FHWRKYGEKNILNSE-FRK---LYYRCGYSDE-RKCQAKKYVQQ----ENNKH-----PPEFRVTLTNEHTCNTV
OsWRKY38    TYAP-YHDG--YQWRKYGQKMIRGNS-FPR---CYYRCTYHQD-HGCPASKHVEQ----HNSED-----PPLFRVIYTNEHTCGTS
OsWRKY41    TASP-YDDG--YQWRKYGQKKINNTN-FPR---SYYRCSYHRE-RRCPAQKHVQQ----RDGDDV----PALHVVVYTHEHTCLQG
OsWRKY40    TPSP-YKDG--YQWRKYGQKNIQDSN-YLR---LYFKCTFSRE-RSCAAKKQVQQ----RDAGE-----PPMFLVTYLNEHTCQQP
OsWRKY9     AQAP-HNDG--HQWRKYGQKWISRAK-HSR---SYYRCANSKV-QGCPATKTVQQMDS--SGNG----TSKLFNVDYYGQHTCRGD
OsWRKY101   AQAP-HNDG--HQWRKYGQKWISRAK-HSR---SYYRCANSKV-QGCPATKTVQQMDS--SGNG----TSKLFNVDYYGQHTCRGD
OsWRKY62    TATP-DYDG--YEWRKYGQKSISKTK-HSR---SYYRCTNQKG-QGCMATKTVQQIENDNSSNS----VVKLYNVDYFGKHTCKFG
OsWRKY10    TPVP-HYDG--HQWRKYGQKHINNSK-HPR---SYYRCTYRQE-EKCKATKTVQQREDLHHANSYNGDHPIMYTVVYYGQHTCCKG
OsWRKY75    TPVP-HYDG--HQWRKYGQKHINNSK-HPR---SYYRCTYRQE-EKCKATKTVQQREDLHHANSYNGDHPVMYTVVYYGQHTCCKG
OsWRKY45    TAVP-HYDG--HQWRKYGQKNINNSN-HQR---SYYRCSYKHE-QNCKATKTVQQ---LDSAG-----ETIMYTVVYYGQHTCKTN
OsWRKY11    TTVP-DFDG--YQWRKYGQKQIEGAL-YPR---SYYRCTNSTN-QGCLAKKTVQR-----NGGG----GAAGYTVAYISEHTCKSI
OsWRKY74    TTVP-DFDG--YQWRKYGQKQIEGAM-YPR---SYYRCTNSTN-QGCLAKKTVQR-----NGGG----GAAGYTVAYISEHTCKSI
AtWRKY38    SPDPIYYDG--YLWRKYGQKSIKKSN-HQR---SYYRCSYNKD-HNCEARKHEQK----IKDN------PPVYRTTYFGHHTCKTE
AtWRKY62    SSTPIYHDG--FLWRKYGQKQIKESE-YQR---SYYKCAYTKD-QNCEAKKQVQK----IQHN------PPLYSTTYFGQHICQLH
AtWRKY63    SPNPRLDDG--FTWRKYGQKTIKTSL-YQR---CYYRCAYAKD-QNCYATKRVQM----IQDS------PPVYRTTYLGQHTCKAF
AtWRKY64    SPTPRPDDG--FTWRKYGQKTIKTSP-YQR---CYYRCTYAKD-QNCNARKRVQM----IQDN------PPVYRTTYLGKHVCKAV
AtWRKY66    SPTPAHIDG--FIWRKYGQKTIKTSP-HQR---WYYRCAYAKD-QNCDATKRVQK----IQDN------PPVYRNTYVGQHACEAP
AtWRKY67    SRTMCPNDG--FTWRKYGQKTIKASA-HKR---CYYRCTYAKD-QNCNATKRVQK----IKDN------PPVYRTTYLGKHVCKAF
OsWRKY19    RVAATLEDG--HVWRKYGQKDIQNSP-YPR---SYYRCTHKLD-QGCGARRQTQRC---EADPSN-------YDITYYGEHTCRDP
OsWRKY71    VVVKNLDDG--QAWRKYGQKEIQNSK-HPK---AYFRCTHKYD-QLCTAQRQVQRC---DDDPAS-------YRVTYIGEHTCRDP
OsWRKY79    VTTKELEDG--RQWRKYGQKHIQDSPNNPR---SYYRCTHRPD-QGCMATKQVQTS---ESNSSE-------FVISYYGEHTCSDP
AtWRKY54    VEAKSSEDR--YAWRKYGQKEILNTT-FPR---SYFRCTHKPT-QGCKATKQVQKQ---DQDSEM-------FQITYIGYHTCTAN
AtWRKY70    IESTILEDA--FSWRKYGQKEILNAK-FPR---SYFRCTHKYT-QGCKATKQVQKV---ELEPKM-------FSITYIGNHTCNTN
OsWRKY68    VRSGTTTDG--FIWRKYGQKEINGCK-HPR---LYYRCAFRG--QGCLATRRVQQSQSQDDPAAA-------FVIAYYGEHTCGGD
OsWRKY93     RKATMDDK--FLWRKYGQKEIKNSK-HPR---FYYRCSYKDD-HGCTATKQVQQSETADDDTASP-----VYIITYFGEHTCRHG
OsWRKY21    QDVGPLDDG--FSWRKYGQKDILGAK-YPR---AYFRCTHRHT-QGCHASKQVQR----ADGDP------LLFDVVYHGDHTCAHG
OsWRKY97    QDVGPLDDG--FSWRKYGQKDILGAK-YPR---AYFRCTHRHT-QGCHASKQVQR----ADGDP------LLFDVVYHGDHTCAHG
```

```
OsWRKY67N  VRSNTEDDG--LSWSKYEQKEILGAK-FPR---AYFRCTHWNTKKGCMATKEVQR----DDGDP------LMFDIVYHGEHTCTQT
OsWRKY66N  RMSYTEDDG--FSWRKYGQKDVEGAM-HPTTQSNYFRCAHKMT-TGCKARKKVQR----TDGDP------LMVDVVYKGVHSCAGV
OsWRKY66C  KDVGPPDDG--YSWKRYGQKNIFGAN-YPR---CYYRCIHKTT-TGCTATKNAQA----TDGDP------LLFDVVYHGEHTCDLQ
OsWRKY67C  VTSVPADDG--YSWRKYGQKNVLGFS-YLR---GYYRCA---T-KGCQASKQVQR----HD-DG------LLFDVTYFGEHTCADQ
OsWRKY82   VENPPVDDG--HSWRKYGQKEILGAK-HPR---GYYRCTHRHS-QGCMATKQVQR----TDEDA------TVFDVIYHGEHTCVHK
OsWRKY100  VENPPVDDG--HSWRKYGQKEILGAK-HPR---GYYRCTHRHS-QGCMATKQVQR----TDEDA------TVFDVIYHGEHTCVHK
OsWRKY53   AEG-PADDG--HSWRKYGQKDILGAK-HPR---GYYRCTHRNT-QGCTATKQVQR----TDDDA------SLFDVVYHGEHTCRPG
AtWRKY41   LEG-PHDDI--FSWRKYGQKDILGAK-FPR---SYYRCTFRNT-QYCWATKQVQR----SDGDP------TIFEVTYRGTHTCSQG
AtWRKY53   LEG-PQDDV--FSWRKYGQKDILGAK-FPR---SYYRCTHRST-QNCWATKQVQR----SDGDA------TVFEVTYRGTHTCSQA
OsWRKY64   LEA-TPDDG--FSWRKYGQKDILGAK-FPR---GYYRCTYRNA-QGCPATKQVQR----SDADL------AVFDVTYQGAHTCHQK
AtWRKY30   VDR-TLDDG--FSWRKYGQKDILGAK-FPR---GYYRCTYRKS-QGCEATKQVQR----SDENQ------MLLEISYRGIHSCSQA
AtWRKY46   QENGSIDDG--HCWRKYGQKEIHGSK-NPR---AYYRCTHRFT-QDCLAVKQVQK----SDTDP------SLFEVKYLGNHTCNNI
OsWRKY70   NTEQPPDDG--YTWRKYGQKDILGSR-YPR---SYYRCTHKNY-YGCEAKKKVQR----LDDDP------FTYEVTYCGNHTCLTS
AtWRKY55   NTDLPPDDN--HTWRKYGQKEILGSR-FPR---AYYRCTHQKL-YNCPAKKQVQR----LNDDP------FTFRVTYRGSHTCYNS
OsWRKY55       MKDDG--WNWIKYGQKDIIGSK-YQR---SYLRCNQMHS-TGCKARKIVEP----SNDDL------NIWLVTYIYEHNHQQR
AtWRKY52     IPAIDEGDLWTWRKYGQKDILGSR-FPR---GYYRCAYKFT-HGCKATKQVQR----SETDS------NMLAITYLSEHNHPRP
OsWRKY87   LELGDRDDS--YPWRKYGQKDILGAR-FAR---SYYRCAQML---GCTARKQVQQ----SDDDP------SRLEITYIGLHTCGGD
```

B

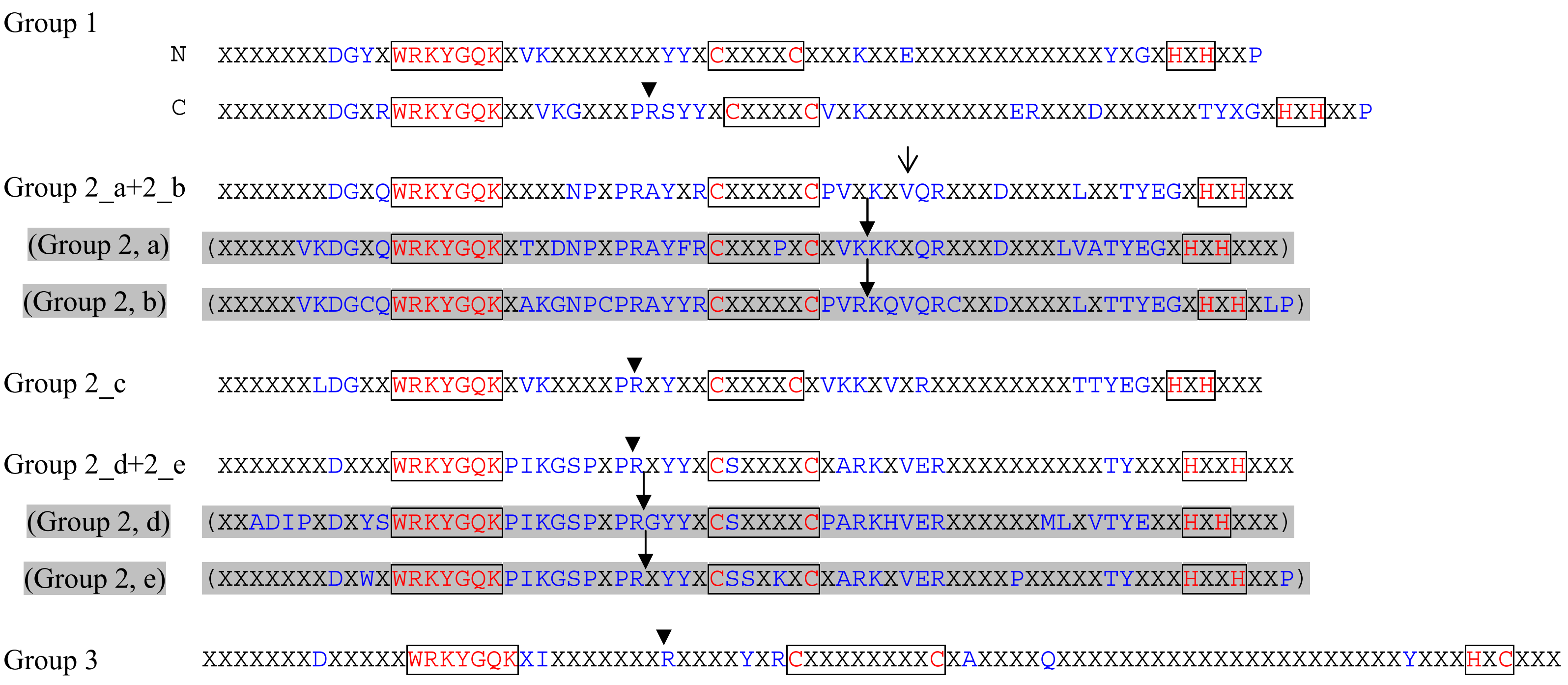

Additional File 2. Multiple alignments, domain classification and sequence conservation patterns of WRKY domains from rice (OsWRKY), Arabidopsis (AtWRKY), the green alga (ChrWRKY), the slime mold (DsWRKY) and *Giardia lamblia* (GlWRKY).

A. Multiple alignments of WRKY domains, which are classified into five groups (Group 1, 2_a+2_b, 2_c, 2_d+2_e, and 3) modified from Eulgem et al. (2000) (see text for details). Sequences in each group are aligned separately. The

heptapeptide WRKYGQK and the zinc-finger motif are in colors (pink for conserved amino acids and blue for substituted amino acids). Other amino acids conserved in all member domains in a group are highlighted in dark yellow.

B. The conservation patterns of WRKY domains. Since subgroups in Group 2, a and b, and d and e from Eulgem et al. (2000) are merged to form two new Groups 2_a+2_b and 2_d+2_e, respectively, the patterns are compared (old classifications highlighted in gray and in parentheses). WRKYGQK and zinc finger motif are highlighted in boxes, and other amino acids conserved in ≥ 90% member domains are in blue. All other amino acids and indels are represented by "X". The conserved intron position is indicated by the arrow (↓, phase 0) or arrowhead (▼, phase 2) in the new classification, while for the old classification the intron position is indicated as by Eulgem et al. (2000). Notice the intron position for Group 2_a+2_b from the present work is different from the previous result by Eulgem et al. (2000).
