# Supplementary material for: The WRKY transcription factor superfamily: its origin in eukaryotes and expansion in plants
Source: BMC Evol Biol. 2005 Jan 3;5:1. doi: 10.1186/1471-2148-5-1 (PMC544883; doi:10.1186/1471-2148-5-1)
Supplement: Additional File 3 — Identified members of the WRKY superfamily in the rice genome [file 1471-2148-5-1-S3.pdf]

Additional File 3 Identified members of the WRKY superfamily in the rice genome

OsWRKY protein	Coding sequence[a]	Chromosome	Group[b]	WRKY domain	
				Name[c]	Pattern[d]
1	387.m00128	10	2_d + 2_e	1	WRKYGQK_X13_C_X5_C_X25_H_X1_H
2	755.m00086, 1463.m00156	3	1C	2C	WRKYGQK_X13_C_X4_C_X25_H_X1_H
	755.m00086, 1463.m00156		1N	2N	WRKYGQK_X13_C_X4_C_X24_H_X1_H
3	755.m00094	3	2_c	3	WRKYGQK_X13_C_X4_C_X25_H_X1_H
4	1903.m00130	6	2_a + 2_b	4	WRKYGQK_X13_C_X5_C_X25_H_X1_H
5	1976.m00181	6	2_a + 2_b	5	WRKYGQK_X13_C_X5_C_X25_H_X1_H
6	1995.m00126	6	2_d + 2_e	6	WRKYGQK_X13_C_X5_C_X25_**H_X2_H**
7	7507.m00115, 8109.m00115	11	3	7	**WRKYGEK**_X13_C_X7_C_X26_H_X1_C
8	7507.m00116, 8109.m00116	11	3	8	**WRKYGEK**_X13_C_X7_C_X26_H_X1_C
9	7507.m00120, 8109.m00120	11	3	9	WRKYGQK_X13_C_X7_C_X29_H_X1_C
10	7507.m00121, 8109.m00121	11	3	10	WRKYGQY_X13_C_X7_C_X35_H_X1_C
11	7507.m00122, 8109.m00122	11	3	11	WRKYGQY_X13_C_X7_C_X26_H_X1_C
12	4935.m00171	2	2_c	12	WRKYGQY_X13_C_X4_C_X25_H_X1_H
13	4971.m00157	9	1C	13	WRKYGQY_X13_C_X4_C_X25_H_X1_H
14	5004.m00127, 6173.m00211, 5016.m00055	9	2_a + 2_b	14	WRKYGQY_X13_C_X5_C_X25_H_X1_H
15	5017.m00157	8	1N	15	**WRICGQK**_X13_C_X4_C_X24_H_X1_H
16	5017.m00158	8	1N	16	**WRMCGQK**_X13_C_X4_C_X24_H_X1_H
17	2471.m00128, 2488.m00119	2	2_a + 2_b	17	WRKYGQK_X13_C_X5_C_X25_H_X1_H
18	2495.m00162	2	2_a + 2_b	18	WRKYGQK_X13_C_X5_C_X25_H_X1_H
19	6966.m00108	3	3	19	WRKYGQK_X13_C_X7_C_X25_H_X1_C
20	5141.m00124, 2802.m00136	1	2_c	20	**WRKYGKK**_X13_C_X4_C_X25_H_X1_H
21	2643.m00178	1	3	21	WRKYGQK_X13_C_X7_C_X25_H_X1_C
22	2673.m00132	1	1C	22C	WRKYGQK_X13_C_X4_C_X25_H_X1_H
	2673.m00132		1N	22N	WRKYGQK_X13_C_X4_C_X24_H_X1_H
23	2688.m00155	1	2_d + 2_e	23	WRKYGQK_X13_C_X5_C_X25_H_X1_H
24	2688.m00165, 2706.m00144	1	2_c	24	WRKYGQK_X13_C_X4_C_X25_H_X1_H
25	6825.m00198	3	unassigned	25	**WKKYGQK**_X13_C_X4_C_X26_H_X1_H
26	2744.m00153	1	2_a + 2_b	26	WRKYGQK_X13_C_X5_C_X25_H_X1_H
27	2744.m00155	1	2_c	27	**WRKYGKK**_X13_C_X4_C_X25_H_X1_H

28	2781.m00198, 4400.m00122	1	2_c	28	WRKYGQK_X13_C_X4_C_X25_H_X1_H
29	2777.m00168, 2702.m00137	1	2_a + 2_b	29	WRKYGQK_X13_C_X5_C_X25_H_X1_H
30	8188.m00107	4	2_d + 2_e	30	WRKYGQK_X13_C_X5_C_X25_H_X1_H
31	2809.m00159, 4409.m00121	1	2_c	31	WRKYGQK_X13_C_X4_C_X25_H_X1_H
32	2802.m00153, 4404.m00122	1	2_a + 2_b	32	WRKYGQK_X13_C_X5_C_X25_H_X1_H
33	2824.m00182	1	2_d + 2_e	33	WRKYGQK_X13_C_X5_C_X25_H_X1_H
34	5372.m00164	4	2_d + 2_e	34	WRKYGQK_X13_C_X5_C_X25_H_X1_H
35	2826.m00124	1	2_c	35	WRKYGQK_X13_C_X4_C_X25_H_X1_H
36	2912.m00098	1	2_c	36	**WRKYGKK**_X13_C_X4_C_X25_H_X1_H
37	8333.m00146	4	1C	37C	WRKYGQK_X13_C_X4_C_X25_H_X1_H
	8333.m00146		1N	37N	WRKYGQK_X13_C_X4_C_X24_H_X1_H
38	2931.m00145	1	3	38	WRKYGQK_X13_C_X7_C_X26_H_X1_C
39	2931.m00148	1	3	39	WRKYGQK_X13_C_X7_C_X26_H_X1_C
40	2931.m00150, 4214.m00162	1	3	40	WRKYGQK_X13_C_X7_C_X26_H_X1_C
41	2931.m00156, 4214.m00168	1	3	41	WRKYGQK_X13_C_X7_C_X27_H_X1_C
42	5434.m00185	4	2_d + 2_e	42	WRKYGQK_X13_C_X5_C_X25_H_X1_H
43	2967.m00101	5	2_a + 2_b	43	WRKYGQK_X13_C_X5_C_X25_H_X1_H
44	2987.m00156, 6195.m00136	5	2_c	44	WRKYGQK_X13_C_X4_C_X25_H_X1_H
45	2988.m00057, 6215.m00159	5	3	45	WRKYGQK_X13_C_X7_C_X27_H_X1_C
46	2981.m00141	5	2_c	46	**WRKYGKK**_X13_C_X4_C_X25_H_X1_H
47	3036.m00114	8	1C	47C	WRKYGQK_X13_C_X4_C_X25_H_X1_H
	3036.m00114		1N	47N	WRKYGQK_X13_C_X4_C_X23_H_X1_H
48	7215.m00096	8	1C	48C	WRKYGQK_X13_C_X4_C_X25_H_X1_H
	7215.m00096		1N	48N	WRKYGQK_X13_C_X4_C_X24_H_X1_H
49	5640.m00114	7	2_c	49	WRKYGQK_X13_C_X4_C_X25_H_X1_H
50	5632.m00190	2	2_d + 2_e	50	WRKYGQK_X13_C_X5_C_X25_H_X1_H
51	3395.m00184	7	1C	51C	WRKYGQK_X13_C_X4_C_X25_H_X1_H
	3395.m00184		1N	51N	WRKYGQK_X13_C_X4_C_X24_H_X1_H
52	5827.m00166	5	2_d + 2_e	52	WRKYGQK_X13_C_X5_C_X25_H_X1_H
53	7393.m00117	8	3	53	WRKYGQK_X13_C_X7_C_X25_H_X1_C
54	3635.m00299	2	2_d + 2_e	54	WRKYGQK_X13_C_X5_C_X25_H_X1_H
55	8164.m00091	7	3	55	**WIKYGQK**_X13_C_X7_C_X25_H_X1_H
56	3687.m00235	8	2_d + 2_e	56	WRKYGQK_X13_C_X5_C_X25_H_X1_H
57	3850.m00168	2	2_d + 2_e	57	WRKYGQK_X13_C_X5_C_X25_H_X1_H

58	6173.m00212	9	2_a + 2_b	58	WRKYGQK_X13_C_X5_C_X25_H_X1_H
59	7019.m00128	7	1C	59C	WRKYGQK_X13_C_X4_C_X25_H_X1_H
	7019.m00128		1N	59N	WRKYGQK_X13_C_X4_C_X24_H_X1_H
60	6195.m00127	5	2_d + 2_e	60	WRKYGQK_X13_C_X5_C_X25_H_X1_H
61	6890.m00221	5	1C	61C	WRKYGQK_X13_C_X4_C_X25_H_X1_H
	6890.m00221		1N	61N	WRKYGQK_X13_C_X4_C_X25_H_X1_H
62	6210.m00168	9	3	62	WRKYGQK_X13_C_X7_C_X31_H_X1_C
63	6231.m00185	5	2_c	63	**WRKYGKK**_X13_C_X4_C_X25_H_X1_H
64	6273.m00136	6	3	64	WRKYGQK_X13_C_X7_C_X25_H_X1_C
65	3999.m00164	10	3	65	**WRKYGEK**_X13_C_X7_C_X26_H_X1_C
66	7350.m00129	11	3C	66C	**WKRYGQK**_X13_C_X7_C_X25_H_X1_C
	7350.m00129		3N	66N	WRKYGQK_X16_C_X7_C_X25_H_X1_C
67	7350.m00122	11	3N	67N	**WSKYEQK**_X13_**C_X8_C**_X25_H_X1_C
	7350.m00122		3C	67C	WRKYGQK_X13_C_X4_C_X24_H_X1_C
68	2931.m00160, 4214.m00172	1	3	68	WRKYGQK_X13_**C_X6_C**_X28_H_X1_C
69	6388.m00159	5	1C	69C	WRKYGQK_X13_C_X4_C_X25_H_X1_H
	6388.m00159		1N	69N	WRKYGQK_X13_C_X4_C_X24_H_X1_H
70	6424.m00214, 6427.m00126	5	3	70	WRKYGQK_X13_C_X7_C_X25_H_X1_C
71	6184.m00147, 6427.m00133	5	3	71	WRKYGQK_X13_C_X7_C_X25_H_X1_C
72	7472.m00138	3	3	72	**WRKYGEK**_X13_C_X7_C_X26_H_X1_C
73	6555.m00148, 7419.m00131	12	1N	73	WRKYGQK_X13_C_X4_C_X24_H_X1_H
74	6642.m00086	12	3	74	WRKYGQK_X13_C_X7_C_X26_H_X1_C
75	6642.m00088	12	3	75	WRKYGQK_X13_C_X7_C_X35_H_X1_C
76	6642.m00092	12	3	76	**WRKYGEK**_X13_C_X7_C_X26_H_X1_C
77	6642.m00094	12	3	77	**WRKYGEK**_X13_C_X7_C_X26_H_X1_C
78	7167.m00153	11	2_c	78	WRKYGQK_X13_C_X4_C_X25_H_X1_H
79	7203.m00119	7	3	79	WRKYGQK_X14_C_X7_C_X25_H_X1_C
80	6678.m00183	3	2_d + 2_e	80	WRKYGQK_X13_C_X5_C_X25_H_X1_H
81	6703.m00204	3	2_d + 2_e	81	WRKYGQK_X13_C_X5_C_X25_H_X1_H
82	5673.m00186	9	3	82	WRKYGQK_X13_C_X7_C_X25_H_X1_C
83	4642.m00123	12	1C	83C	WRKYGQK_X13_C_X4_C_X25_H_X1_H
	4642.m00123		1N	83N	WRKYGQK_X13_C_X4_C_X24_H_X1_H
84	4697.m00181	1	2_d + 2_e	84	WRKYGQK_X13_C_X5_C_X25_H_X1_H
85	4726.m00203, 4755.m00202	5	2_c	85	WRKYGQK_X13_C_X4_C_X25_H_X1_H

86	4755.m00215	5	2_a + 2_b	86	WRKYGQK_X13_C_X5_C_X25_H_X1_H
87	7286.m00185	7	3	87	WRKYGQK_X13_C_X5_C_X25_H_X1_C
88	2750.m00167	1	2_a + 2_b	88	WRKYGQK_X13_C_X5_C_X14_H_X1_H
89	4512.m00084	12	2_d + 2_e	89	WRKYGQK_X13_C_X5_C_X25_H_X1_H
90	2758.m00136, 2713.m00101	1	2_c	90	WRKYGQK_X13_C_X4_C_X25_H_X1_H
91	NP164417 (mRNA)	unknown	1C	91	WRKYGQK_X13_C_X4_C_X25_H_X1_H
92	NP183745	1	3	92	**WRKYSEK**_X13_C_X7_C_X25_H_X1_H
93	7354.m00183	5	3	93	WRKYGQK_X13_C_X7_C_X30_H_X1_C
94	7354.m00184	5	3	94	**WRKYGEK**_X13_C_X7_C_X26_H_X1_C
95	7354.m00185	5	unsigned	95	WRKYGQK_X13_C_X5_C_X27_H_X1_C
96	34.m00132	unknown	2_a + 2_b	96	WRKYGQK_X13_C_X5_C_X25_H_X1_H
97	2789.m00133	1	3	97	WRKYGQK_X13_C_X7_C_X25_H_X1_C
98	2940.m00157	2	2_c	98	WRKYGQY_X13_C_X4_C_X25_H_X1_H
99	2943.m00201	2	2_c	99	WRKYGQY_X13_C_X4_C_X25_H_X1_H
100	4510.m00122	9	3	100	WRKYGQK_X13_C_X7_C_X25_H_X1_C
101	6642.m00089	12	3	101	WRKYGQK_X13_C_X7_C_X29_H_X1_C
102	6739.m00147	3	2_d + 2_e	102	WRKYGQK_X13_C_X5_C_X25_H_X1_H
103	7142.m00128	7	1C	103C	WRKYGQK_X13_C_X4_C_X25_H_X1_H
	7142.m00128		1N	103N	WRKYGQK_X13_C_X4_C_X24_H_X1_H
104	7220.m00162	11	1N	104	WRKYGQK_X13_C_X4_C_X24_H_X1_H
105	4214.m00160	1	3	105	WRKYGQK_X13_C_X7_C_X26_H_X1_C

[a]CDS accessions of TIGR's OSA1

[b]Phylogenetic classification according to Eulgem et al. (2000) with modifications (see text). Postfix of N or C to the group number indicates N- or C-terminal domain for two-domain proteins.

[c]The WRKY domain name is the same as its protein name except for two WRKY domain-containing proteins. In this case, the protein name is followed by N or C to indicate an N- or C-terminal domain.

[d]Variants of conserved WRKYGQK peptide and the zinc-finger motifs are bolded.
