# Supplementary material for: The WRKY transcription factor superfamily: its origin in eukaryotes and expansion in plants
Source: BMC Evol Biol. 2005 Jan 3;5:1. doi: 10.1186/1471-2148-5-1 (PMC544883; doi:10.1186/1471-2148-5-1)
Supplement: Additional File 4 — Survey of WRKY genes from ESTs or their assembled gene indices for 19 plants and the phylogenetic classification of the genes [file 1471-2148-5-1-S4.pdf]

Additional File 4 Survey of WRKY genes from ESTs or their assembled gene indices for 19 plants and the phylogenetic classification of the genes

Common name	Scientific name	Source sequence set	Release date	Total ESTs	WRKY			Classification of WRKY domains[a]					
					unique genes	ESTs	% EST (WRKY/Total)	1C	1N	2a + 2b	2c	2d + 2e	3
Cotton	*Gossypium* spp.	CGI5	8/14/2003	53,970	20	27	0.05	7	2	4	3	4	2
Soybean	*Glycine max*	GmGI11	8/21/2003	334,730	109	627	0.1873	22	13	21	19	27	12
Sunflower	*Helianthus annuus*	HaGI3	8/18/2003	59,713	13	57	0.0955	4	0	3	5	0	2
Barley	*Hordeum vulgare*	HvGI7	8/20/2003	343,206	53	211	0.0615	4	8	6	9	6	21
Tomato	*Lycopersicon esculentum*	LGI9	4/17/2003	156,645	51	555	0.3543	9	8	10	6	10	11
Lotus	*Lotus japonicus*	LjGI2	4/21/2003	33,090	13	29	0.0876	3	1	1	1	5	2
Lettuce	*Lactuca sativa*	LsGI1	10/1/2002	68,178	18	54	0.0792	2	3	2	4	4	4
Ice plant	*Mesembryanthemum crystallinum*	McGI4	4/23/2003	25,840	1	1	0.0039	0	0	0	1	0	0
Medicago	*Medicago truncatula*	MtGI7	5/7/2003	189,919	62	430	0.2264	9	9	9	11	17	10
Onion	*Allium cepa*	OnGI1	9/4/2003	19,629	17	28	0.1426	3	4	4	1	4	3
Pine	*Pinus* spp.	PGI3	8/20/2003	81,802	4	7	0.0086	3	0	0	0	1	0
Rye	*Secale cereale*	RyeGI2	8/5/2001	8,971	5	5	0.0557	0	2	0	1	1	1
Sorghum	*Sorghum bicolor*	SbGI6	8/20/2003	130,897	27	75	0.0573	3	5	10	3	0	7
Potato	*Solanum tuberosum*	StGI7	4/24/2003	94,929	39	134	0.1412	6	2	9	6	11	7
Wheat	*Triticum aestivum*	TaGI7	8/20/2003	494,195	68	229	0.0463	6	7	6	14	18	17
Grape	*Vitis vinifera*	VvGI3	8/18/2003	109,141	24	105	0.0962	8	1	1	5	7	3
Maize	*Zea mays*	ZmGI13	8/27/2003	364,267	51	184	0.0505	12	5	5	14	10	6
Moss	*Physcomitrella patens*	ESTs	11/3/2003	102,800	6	10	0.0097	1	0	0	2	3	0
Fern	*Ceratopteris richardii*	ESTs	11/3/2003	3,634	1	1	0.0275	1	0	0	0	0	0

[a]WRKY domains are phylogenetically classified according to Eulgem et al. (2000) with modifications (see text). For Group 1, N or C indicates N- or C-terminal domain.
